# Agri-Food Side-Stream Inclusion in The Diet of *Alphitobius Diaperinus*. Part 2: Impact on Larvae Composition

**DOI:** 10.3390/insects11030190

**Published:** 2020-03-17

**Authors:** Lise Soetemans, Natasja Gianotten, Leen Bastiaens

**Affiliations:** 1 Flemish Institute for Technological Research (VITO), Boeretang 200, 2400 Mol, Belgium; lise.soetemans@vito.be; 2 Department of Food and Drug, University of Parma, 43121 Parma, Italy; 3 Protifarm, Harderwijkerweg 141B, 3852 AB Ermelo, The Netherlands; n.gianotten@Protifarm.com

**Keywords:** lesser mealworm, correlation, proteins, lipids, amino acids, fatty acids, larval composition, composition of side streams, nutritional value

## Abstract

Insects are gaining interest as an alternative protein source for feed/food purposes. Although the lesser mealworm (LM) is commercially produced for human consumption, published data on its nutrient composition is scarce. This study reports on LM larvae reared on 18 different diets composed of side-streams to (1) determine the nutritional composition of the larvae and (2) study the effect of dietary changes on the larval nutrient composition. The LM larvae proved to be of good nutritional value with essential amino acids profiles comparable with that of beef and linoleic acid (C18:2) was the most dominant essential fatty acids in the larvae. The side-stream based diets varied on dry matter basis in protein (16–34%) and lipid content (2–19%). The nutrient content of the larvae reared on diets that supported good growth ranged between 37% and 49% of protein, 22% and 26% of lipid and 4% to 6% of chitin on dry matter basis. No significant correlations were identified between the larval protein or lipid content and that of the diet, but it was found between the diet nutrients and larval growth. Based on larval growth data and economic considerations, diets composed of wheat middlings with a 10–15% inclusion of rapeseed meal were identified as suitable feed for LM. Highest larval yields were obtained with diets containing 15–22% of proteins and 5–10% of lipids.

## 1. Introduction

Growing concerns about future protein shortage are inducing a search for alternative protein sources. Especially for countries and continents (like Europe) where protein-rich ingredients are mostly imported, sustainable and local protein sources become increasingly important [1,2]. Insects offer an opportunity since they are rich in proteins (ranging from 13% to 77% on dry matter (DM) basis) and have been, in some parts of the world, part of the human diets for centuries [3]. For instance, the larvae of the black soldier fly (BSFL, *Hermetia illucens*), common housefly larvae (*Musca domestica*), silkworms (*Bombyx mori)* and yellow mealworms (YM, *Tenebrio molitor*) have been described as promising species for industrial food or feed applications and have a protein content of 56%, 62%, 54% and 52% on DM basis, respectively [3,4,5]. Recently, a considerable amount of literature was published on the BSFL and YM, but information on the lesser mealworm (LM, *Alphitobius diaperinus)* is still rather limited. Nevertheless, the latter species is reported to be rich in proteins, is suitable for human consumption and has in comparison to the YM a shorter development time (66 days versus 117 days till 50% pupation) [4,6]. Van Broekhoven et al. (2015) even found a higher protein content of the LM larvae compared to the YM larvae when reared on the same diet [6]. The rearing of insects is commonly performed with grain-based diets. However, food byproducts are also being introduced in the diet to avoid future competition for grains and to obtain a more low-cost diet. In addition, by using (underspent) side-streams, side-stream nutrients can be recycled back in the market as insect proteins. However, the impact of the feed on the composition of the insect is a point of attention. It has been reported that for some insect species the insect composition can be altered by the diet. Research on the BSFL, for example, proved that the lipid content and fatty acid profile were influenced by the diet [7,8,9]. This could generate opportunities to tune the nutritional value of the insects for example in essential amino acid composition or fatty acid profile [10]. Published data on the compositional changes in mealworms are scarce and sometimes contradictory. Some studies observed no changes in larval lipid content when providing different diets [9,11] whereas Van Broekhoven et al. (2015) did measure a variable larval lipid content with different diets. However, a direct connection between the dietary lipid content was not found as two diets with similar lipid content provided larvae with a significantly different amount of lipids [6]. In addition, Dreassi et al. (2017) and Van Broekhoven et al. (2015), measured differences in the fatty acid profile when the YM was reared on different diets. These findings were partially confirmed by Oonincx et al. (2015) who found only a slight variation in fatty acids C16:0 and C18:2 and stated that the larval fatty acid did change over different rearing diets but the changes did not follow the fatty acid profile changes of the diet [9]. In respect to the protein content, a rather constant larval protein content was measured over different diets (between 62% and 65% for the LM [6] and between 44% and 54% for the YM [9]) when the diet changed in side-stream ratios of spent grains, beer yeast, cookie remains, potato peelings, beet molasses, bread and maize distillers dried grains with solubles (DDGS).

The LM is among the seven insect species on a list that allows the use of their proteins in feed for aquaculture animals (Regulation No 2017/893). Yet, published data on the nutrient composition of the LM larvae is scarce, as well as literature on the dietary effects on the composition of LM. The current study is part of a larger study on the impact of agri-food side-stream inclusion in the diet of the LM larvae. Firstly, the impact of 29 diets on the LM larval growth was studied and reported by Gianotten et al. [12]. The larvae were found able to grow on all diets, but differences in growth were observed. The current study (part 2) reports on the composition of different diets and of the larvae grown on these diets. More specifically, a selection of 18 out of the 29 diets and the corresponding larvae were studied in terms of nutrient composition. The aim was to 1) determine the major nutrient composition of the LM larvae and 2) the correlation between the dietary composition and the larval composition, and 3) to evaluate if there is a correlation between the larval yield and the dietary composition.

## 2. Materials and Methods

### 2.1. Insects and Diets

Lesser mealworm larvae that were derived from the experimental setup of Gianotten et al. were reared on different side-stream compositions as described by Gianotten et al. [12]. Briefly, larvae were reared on semi-industrial scale (about 45,000 larvae per tray) under optimal rearing conditions (between 28 and 32 °C and a humidity above 60%). First instar larvae (freshly hatched) were immediately reared on the selected diets for 28 days (standard development time for obtaining commercially mature larvae on a standard diet). For each diet, the rearing was performed in eightfold. After separating the larvae from the residue by sieving, a subsample (about 250 g of larvae per tray) of every tray (8 trays per diet) was taken and mixed. These mixed larvae samples were stored at −20 °C until they were freeze-dried. After freeze-drying, three separate subsamples were taken for analysis. The selected diets contained mixtures of wheat middlings, rice bran, rapeseed meal, distillers dried grains with solubles (DDGS) and corn gluten feed and carrot or brewery grains (BG) as a source of moisture. Carrots were assumed not to contain considerable amounts of nutrients supporting the growth of the LM, while BG do contain other nutrients [13]. Details on the selected different side-stream mixtures from the study of Gianotten et al. [12] and the relative larval yield (compared to diet 2) obtained are summarized in Table 1.

### 2.2. Composition Analysis

Freeze-dried LM larvae and the side-streams used were subjected to a set of analyses to determine their composition. The dry matter (DM) was determined after drying the samples at 105 °C for 48 h and ash content after mineralization at 550 °C for 6 h. All composition data for the larvae and feed are reported on a DM basis. Soxhlet extractions with diethyl ether for 6 h were performed to determine the lipid content gravimetrically (g lipid/100 g DM). The fatty acids profile was measured on Soxhlet extract by gas chromatography flame ionization detection (GC-FID) after methylation with NaOH/MeOH and H2SO4/MeOH (pretreatment according to ISO 12966-2:2011 and AOCS Ce 1b-89). Analyses with GC-FID were performed with a FAMEWAX column (30 m × 0.32 mm, 0.25 µm df) at a constant flow rate of 1 mL/min helium. The split/splitless injector was set at a temperature of 245 °C and the split flow at 75 mL/min. The FID detector was set at 250 °C. The internal standard was methyl heptadecanoate and the standards lauric acid and oleic acid (50–20000 µg/g) underwent the same methylation pretreatment as the samples. Amino acids were determined on defatted samples after acid hydrolysis with phenol-HCl (6 N) for 23 h at 110 °C under a nitrogen environment (pretreatment according to ISO 13903:2005). Amino acids were subsequently analyzed by high-performance anion exchange chromatography with pulsed amperometric detection in Chromeleon software. The columns Dionex AminoPac PA-10 (2 nm × 250 nm) and Dionex AminoPac PA-10 Guard (2 mm × 50 mm) were used at 30 °C. The mobile phases (0.250 mL/min) consisted of (A) Milli-Q water, (B) 250 mM NaOH, (C) 1M NaOAc, (D) 0.1 M Acetic acid; gradient: 76% Eluent A and 24% B (0–2 min), 64% eluent A and 36% eluent B (2–11 min), 40% eluent A, 40% eluent C and 20% eluent A (11–47 min), 100% eluent D (47.1–49.1 min), 20% eluent A and 80% eluent B (49.2–51.2 min), 76% eluent A and 24% eluent B (51.3–76 min). Tryptophan was not measured given its notorious tendency to be degraded during acid hydrolysis. During the acid hydrolysis, asparagine and glutamine were converted to aspartic acid and glutamic acid, respectively. The acid hydrolysis may degrade methionine and cysteine and a nitrogen environment was applied to minimal degradation of methionine. However, these amino acids were measured in a less optimal environment and the results should be interpreted with care. Nevertheless, the same pretreatment was applied in order to enable comparisons between samples. The total protein content was calculated as the sum of mmols of the individual anhydrous amino acid residues (subtracting one molecule of water from the molecular weight of each amino acid) per g sample and were reported as % protein (g protein/100 g DM). In this study, the term ‘essential amino acids’ refers to amino acids that are essential for humans or insects and will be specified. Quantification of the chitin content (g chitin/100 g DM) was performed as described by D’Hondt et al. [14]. Briefly, chitin was hydrolyzed with 6 M HCl for 6 h at 110 °C. The released glucosamine was subsequently quantified by LC–MS (Waters UPLC BEH HILIC 2.1 mm × 100 mm, 1.7 µm column at 40 °C, isothermal gradient elution using water with (A) 20 mM ammoniumformiate and 0.1% formic acid and (B) acetonitrile with 0.1% formic acid with gradient settings: 5–25% A (0–3 min), 25% A (3–4 min), 25–5% A (4–4.1 min) and 5% A (4.1–7 min)). Quantification was performed against a set of standard solutions.

### 2.3. Statistical Analysis

All analyses were performed in triplicate unless stated otherwise. The data were expressed as the averages with the standard deviation (SD) and were statistically processed by one-way analysis of variance (ANOVA, *p*< 0.05) followed by a Tukey post hoc test by using IBM SPSS software. The Shapiro–Wilk test was used to control the data on a normally distribution (*p* < 0.05) and Levene’s test was used to judge the variance of the population (*p* < 0.05). When these terms were not met, a Kruskal–Wallis analysis was performed with a pairwise comparison, and significant values were adjusted by Bonferroni correction. In addition, a simple linear regression analysis was performed with the composition of the diet as the predictor and the composition of the larvae as the criterion (*p*<0.05) by using IBM SPSS software to evaluate linear correlations. The assumption of normality of the residuals, the linear relationship between the variables and the homoscedasticity were checked by evaluating the scatterplots, the normal predicted probability plots and the residuals scatterplots. The variance inflation factor value was evaluated (<10) for the absence of multicollinearity. The Pearson correlation coefficient was calculated to evaluate the linear correlation.

## 3. Results and Discussion

### 3.1. Composition of the Side-Streams and Insects Diets

The composition of all feed ingredients (wheat middlings, rice bran, corn gluten feed, rapeseed meal, DDGS and BG) were characterized in terms of protein and lipid content, amino acids profile and fatty acid profile (Appendix A: Table A1). DDGS and rapeseed meal were the most protein-rich (30% protein) ingredients, while rice bran contained the highest lipid content (19%) followed by DDGS (14%). Wheat middlings, rapeseed meal, and corn gluten feed contained a very low lipid content (≤ 5). The amino acid profile of all ingredients revealed that arginine and glutamate were most dominant. Yellow mealworms require the same nine essential amino acids (EAA) as humans, plus arginine, and the same was presumed for the lesser mealworm [15,16]. Rapeseed meal and DDGS were most nutritional in terms of the presence of EAA for insects (188 g/kg and 202 g/kg, respectively). The fatty acid profiles indicated that C16:0, C18:1 and C18:2 were mainly present for all side-streams. Since no literature was found on the essential fatty acids (EFA) for insects, the fatty acids essential for human consumption were also considered essential for insects in the current study. Rice bran contained more lipids than DDGS but a better nutritional value in terms of the sum of all EFA (61 g/kg versus 55 g/kg, respectively) was found for DDGS.

**Figure 1:**
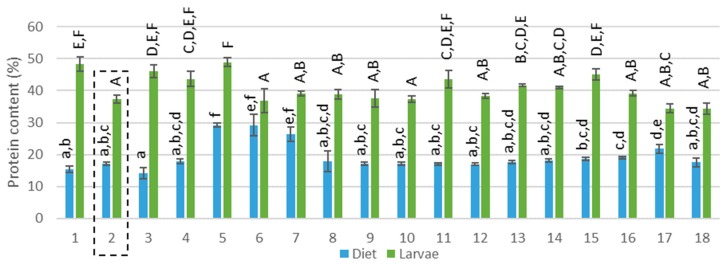
Protein content (% on dry matter basis) of the diets and corresponding larvae. Diet 2 (reference diet) is framed, capital letters represent the pairwise comparison after the Kruskal–Wallis analysis performed on the protein content of the larvae while regular letters represent the pairwise comparison after the Kruskal–Wallis analysis performed in the protein content of the diets.

**Figure 2:**
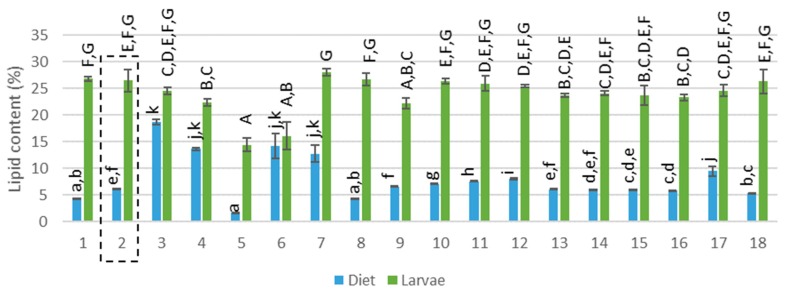
Lipid content (% on dry matter basis) of the diet and corresponding larvae. Diet 2 (reference diet) is framed, capital letters represent the pairwise comparison after the Kruskal–Wallis analysis performed on the lipid content of the larvae while regular letters represent the pairwise comparison after the Kruskal–Wallis analysis performed in the lipid content of the diets.

The compositions of the mixed diets were theoretically calculated based on the proportion of each feed ingredient given (see Appendix A: Table A2 and Table A3). The protein content in the diets ranged between 14% and 29% (see Figure 1, F(17,36) = 28.107; *p*<0.001) and the lipid content between 2% and 19% (see Figure 2, F(17,36) = 103.816; *p*>0.001). Considering the presence of EAA for insects, diet 5 (100% rapeseed), 6 and 7 (100% DDGS) provided the most nutritional diet (> 170 g/kg EAA) whereas diet 1,2,9–11 and 18 were the poorest (< 100 g/kg EAA). In terms of EFA, C18:2 and C18:3 were present in high amounts in all diets except for a low amount of C18:2 for diet 5 (100% rapeseed meal). The sum of all EFA was between 2.3 and 61.0 g/kg where diets 3, 4, 6, 7 and 17 were rich in EFA and diets 1, 5, 8 and 18 were poor. Brewery grains, providing moisture but also nutrients, had the highest C18:3 concentration and was presumed to be the main provider of this fatty acid in all diets. In conclusion, the 18 diets that were tested differed in origin (different side-streams) and also in nutritional composition. Diet 2, containing wheat middlings mixed with BG, was pointed out by Gianotten et al. [12] as a reference diet that provided a good yield. This diet will also be addressed as a reference diet in the current study. Diets 5–7 and 17 contained a different protein content compared to the reference diet and diets 1,3–8, 10–12 and 16–18 had a different lipid content.

### 3.2. Composition of the Larvae

The LM larvae reared on 18 different diets, had a DM content between 23% and 33%. The protein content of the larvae ranged between 37% and 49% (see Figure 1, F(17,34) = 14.338, *p*>0.001 and Appendix A: Table A4), the lipid content between 14% and 28% (see Figure 2 F(17,36) = 23.551; *p*>0.001 and Appendix A: Table A5) and the ash and chitin content between 4% and 7% and between 4.2% and 6.2%, respectively. This composition is comparable to data found in the literature. Yi et al. (2013) determined a dry matter of 35.2% while Van Broekhoven et al. (2015) reported a value between 30% and 33% for different rearing conditions [6,17]. The protein content in literature for the LM larvae ranges from 48% [18] to 65% [4,6,19,20]. This broad range can be explained by a different larval age [21] or a different method of data processing or analytical method. The protein content is often determined based on nitrogen measurements where a nitrogen–protein conversion factor of 6.25 is used. However, Janssen et al. (2017) and Mishyna et al. (2019) have pointed out that this conversion factor leads to an overestimation due to the chitin (also contains nitrogen) present in insects [18,22] Janssen et al. (2017) calculated a conversion factor of 4.86 for the LM and found a protein content of 48.8% [18]. This result is more in line with the protein content measured in the current study (where the protein content was calculated based on the total amino acid analysis). However, the amino acid profile does not contain tryptophan and thus may lead to a slight underestimation. Several diets (diets 1, 3–5, 11 and 15) were able to significantly increase the protein content of the larvae compared to the reference diet (see Figure 1). In view of the lipid content, published data indicate a lipid content between 13% and 25% for the LM [4,6,15,22], which is matching well with the findings of the current study. All rearing trials were ended after 28 days and for some conditions a low larval yield was observed, which means that the larval weight (mg per larvae) was low. This could imply that the larvae of these trials were not yet mature and thus in another larval stage. As the composition, and especially the lipid content, of the larvae can differ between development [23,24] a lower larval yield corresponded in some cases (diets 5 and 6) with a lower lipid content, explaining the large variance in lipid content. Yet, diets 3 and 4 also resulted in a low larval yield, but an average lipid content was measured. The lipid content of the larvae was for all diets similar to the reference diet or lower (see Figure 2). Janssen et al. (2017) found a similar chitin content (between 4.4% and 9.1%) for the LM larvae [18] and an ash content of 4.1% for the LM larvae was reported by Bosh et al. (2014) [4]. Carbohydrates, besides chitin, are also present in insects, for instance about 10% was reported by Janssen et al. (2017) for the LM larvae [18]. Non-chitin carbohydrates as well as tryptophan were not quantified in the current study and may explain the incomplete mass balance.

**Figure 3:**
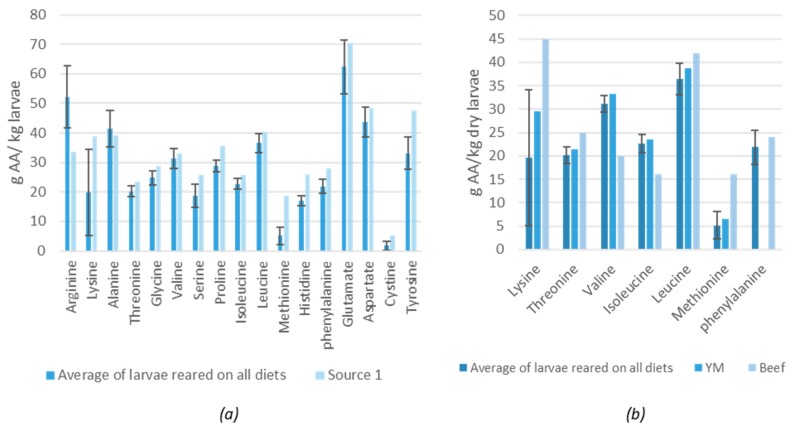
(**a**) Average amino acid profile of lesser mealworm (LM) larvae reared on the diets in the current study compared to data found in literature (Source 1: [19]). (**b**) Comparison of the essential amino acids in LM larvae (measured in the current study) with yellow mealworm (YM) larvae [27] and beef [3], AA = amino acid, YM = yellow mealworm.

The most dominant amino acids in the larvae (higher than 32 g/kg) were glutamate, arginine, aspartate, alanine, leucine and tyrosine (Figure 3a). The diets also contained a high concentration of arginine, leucine, glutamate and aspartate but not for tyrosine and alanine. This observation suggests that the latter compounds are of importance for the larvae and were concentrated or synthesized by the larvae. In fact, tyrosine is known to be involved in the production of melanin that is employed for cuticular hardening, wound healing and innate immune responses with insects [25,26] The average amino acid profile of the larvae reared on the different diets was similar to the data reported by Despins and Axtell (1995) [19]. Janssen et al. (2017) also found the same amino acids (expect for arginine) to be dominant in the LM larvae just as Bosch et al. (2016) for the YM [18,27]. Figure 3b shows the EAA profile of the YM and beef for human consumption. A similar profile between the LM larvae (data of the current study) and the YM larvae was found. Van Huis et al. (2013) did report a much higher leucine content for the YM larvae but Bosch et al. (2016) and Heidari-Parsa et al. (2018) reported similar leucine contents as the LM larvae in the current study [3,26,27]. When comparing the profile of the LM larvae with beef, lower values of lysine and methionine were observed for the LM larvae, but higher for valine and isoleucine.

In respect to fatty acid profiles, the most dominant fatty acids of the larvae were C18:2, C18:1 and C16:0 (>10g/kg) and to a lesser extent C18:0, C18:3, C14:0 and C16:1 (>1g/kg; see Appendix A: Table A5), which is in line with the findings of Van Broekhoven et al. (2015) and Tzompa-Sosa et al. (2014) [6,20]. When comparing to the YM, the same dominant fatty acids were found [9,27,28,29]. Essential fatty acids’ linoleic acid (C18:2) and linolenic acid (C18:3) represented about 22–41% and 0.6–2.6% of the total fatty acids measured. This corresponds well with other published data on the LM (17–36% for C18:2 and 0.4–0.7% for C18:3 [6]) and the YM (23–31% for C18:3 and 0.6–1.1% for C18:3 [23]). The total saturated fatty acids (SFA) ranged between 28 and 75 g/kg, the total monounsaturated fatty acids (MUFA) between 39 and 88 g/kg and the total polyunsaturated fatty acids (PUFA) between 23 and 91 g/kg. Diets 6 and 7 (both containing 100% DDGS) resulted in larvae with an elevated amount of C18:3 and the diets that contained a lower concentration of C18:3 also resulted in larvae with a small decrease in C18:2.

### 3.3. Correlations between the Diet Composition and the Larvae Composition

When comparing the protein content of the larvae with the protein content of the diets (Figure 1), it is clear that the larvae concentrate the protein. This is reflected in the high larval protein content in comparison to the content of the diets. This was also reported by Stull et al. (2019) who measured a high protein content in YM larvae bred on the low-nutrient and low-protein feed of stover [30]. In addition, it seems that variations in dietary protein amount were not translated into the larval biomass. For instance, diets 7 and 8 had a decreasing protein content while the larvae exhibited a similar protein content. The Pearson correlation coefficient (R) indicated that no linear correlation was found between the protein content of the diet and that of the larvae (R = 0.039, F(1,52) = 0.076, *p* = 0.784, R² = 0.002). Van Broekhoven et al. (2015) also found no clear impact of the dietary protein content that ranged from 12% to 39% and the larval content that remained rather constant (32.2% ± 1.6%) [6].

A variation in larval amino acid concentration was observed over the different diets (for example, 34–56 g/kg for alanine, 7–32 g/kg for serine and 21–40 g/kg for tyrosine). Most amino acids did not have a significant correlation with the dietary concentrations (R<0.3, *p*> 0.05, see Table 2). This was expected since non-essential amino acids in the diets are digested, absorbed and metabolized to insect proteins but five out of the eight EAA also did not show a significant correction. Only EAA threonine (R = 0.38, F(1,52) = 7.494, *p* = 0.009, R² = 0.143), leucine (R = 0.51, F(1, 52) = 15.424, *p*< 0.001, R² = 0.255) and isoleucine (R = 0.30, F(1,52) = 4.553, *p* = 0.038, R² = 0.092) in the diet had a slight impact on the larval concentrations. Furthermore, non-essential amino acids alanine (R = 0.29, F(1, 52) = 4.238, *p* = 0.045, R² = 0.086) and glutamate (R = 0.39, F(1, 52) = 8.287, *p* = 0.006, R²= 0.156) in the diet also had a significant impact on the larval concentration variance. Nevertheless, R² is no greater than 25% and thus only a slight impact is expected. No significant impact was measured for methionine, although the concentration of methionine varied greatly (1–9 g/kg) even though the dietary concentration was constant. These results are in line with Ramos-Elorduy et al. (2002), who also found varying amino acids concentrations when YM were reared on different diets [31]. In addition, the larvae were not starved before freezing and thus some residual amino acids in the guts could also explain the larger variance. Insects, like humans, require EAA that are needed for protein build up [15,16]. In the current study, no clear impact of the EAA concentration in the insect diets was detected on the corresponding larval protein content. For example, diets 6–8 had a decreased EAA concentration but no effect of this decrease was translated in the larval protein content, which stayed constant. This can indicate that the necessary amounts of the EAA were already present in diet 8 (lowest EAA containing diet). On the other hand, diet 15 had a lower total EAA concentration compared to diet 17 but the larvae generated by diet 15 also had a significantly higher protein content. In this case, it could be that the digestibility of the dietary EAA was different since the diet ingredients were different and the digestibility was better for proteins in diet 15. Tryptophan, an EAA for insects was not measured and could also explain some differences.

In terms of lipid content, the data in Figure 2 indicate that the larvae also concentrated or metabolized lipids. This conclusion is based on a visually increased lipid content of the larvae compared to the diets they were reared on. Fluctuations in the dietary lipid content were not translated into fluctuation of lipids in the larvae, which was confirmed by the very low Pearson correlation coefficient (R = 0.011, F(1.52) = 0.006, *p* = 0.937). Compared to the literature, also no significant changes in larval lipid content of the YM were detected with a dietary lipid content between 0.5% and 9.3% [11] and Oonincx et al. (2015) stated that, for YM, the fatty acid profile of insects does not reflect that of the diet [9]. Behmer (2009) stated that insects regulate their nutrient intake when the opportunity is given [32]. To the author’s vision, this statement is applicable in optimal conditions, where all required nutrients are abundantly available. However, some restrictions would impact nutrient intake. For example, when an insufficient concentration of an important dietary compound is available, the insect switches to a ‘survival mode’ where the intake of other nutrients will be increased [6,33,34]. For example, during a lipid shortage, carbohydrates are converted to lipids by insects [6,35,36]. Carbohydrates were not measured in this study, but wheat middlings, the basal ingredient in the mixed diets, is known to be rich in carbohydrates (sum of starch and sugar 28.1%, Appendix A: Table A1). Diets 5 (100% rapeseed meal) and diet 6 (100% DDGS) generated larvae with a significantly lower lipid content, which corresponded with a low carbohydrate and lipid content in both substrates. Diet 7, however, consisted also of 100% DDGS but the moisture source BG provided extra carbohydrates (6%) and lipids (10%), resulting in larvae with an average lipid content. The study suggests that carbohydrates in the diets may have played a role in stabilizing the larval lipid content. On the other hand, rice bran is rich in lipids as well as in carbohydrates, yet, the surplus of the two nutrients did not result in higher lipid-containing larvae.

For most fatty acids, no significant correlations (R< 0.5, see Appendix A: Table 2) were observed. This can be explained by the fact that most fatty acids are metabolized by the organism. The fatty acid C20:4, for instance, was not detected in the diet but was found in the larvae. For insects, and the LM, in particular, no literature was found on the designation of insect essential fatty acids. According to the current study, five fatty acids were found that showed a significant, slight linear correlation. Fatty acids C18:3 (F(1,52) = 17.985, *p*< 0.001, R² = 0.257) and C20:2 (F(1,52) = 15.057, *p*< 0.001, R² = 0.251) had a Pearson correlation factor of 0.5 indicating a positive correlation of the fatty acid concentration in the diet with the concentration in the larvae. Fatty acids C18:1 (R = 0.38, F(1,52) = 8.783, *p* = 0.005, R² = 0.144), C18:2 (R = 0.32, F(1,52) = 5.947, *p* = 0.018, R² = 0.103) and C20:0 (R = 0.332, F(1,52) = 6.450, *p* = 0.014, R² = 0.11) also had a significant correlation. Again, only a maximum of 25% of the variance of the larval concentration could be explained by the dietary concentration. However, an influence of the carbohydrates could not be excluded, as well as a possible presence of lipids and fatty acid in the gut, which are probably responsible for a large range in other larval fatty acid concentration (for example C14:1 ranging from 7 to 176 mg/ kg). Van Broekhoven et al. (2015) found similar results and concluded that the fatty acid composition in the diet influenced the larval fatty acid composition but not in the same trend and that physiological regulation of the larval fatty acid composition takes place [6]. The same statement was reported by Dreassi et al. (2017) [11]. In conclusion, no or only slight linear correlations were found between the fatty acid composition of the diet and the larvae.

### 3.4. Correlations between the Diet Composition and the Larvae Yield

**Figure 4:**
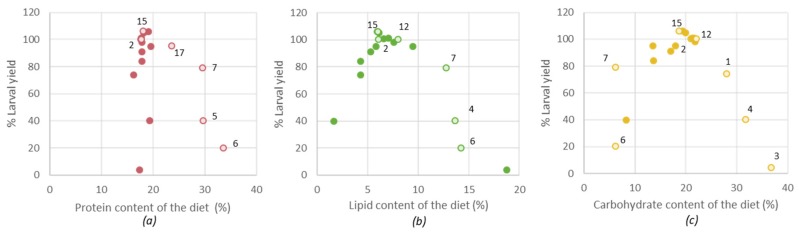
The relation between the dietary nutrient component and the larval yield for (**a**) the protein content, (**b**) the lipid content, (**c**) the carbohydrate content. Hollow bullets were indicated by their diet number. Diet 2 = reference diet.

Gianotten et al. described that higher larval yields were obtained for certain diets that were also included in the current study [12]. Figure 4 summarizes the relationship between the % larval yield versus proteins, lipids and the theoretically calculated carbohydrates. The data suggest that, for the diets evaluated in the current study, optimum larval yield was achieved with 17–22% dietary protein, 6–8% dietary lipid and 18–20% dietary carbohydrates. It must be emphasized that the diets were not optimized for maximal growth, but for the use of specific side-streams, and that other nutrients (other than proteins, lipids and carbohydrates) can also influence the growth of the larvae. Even though the larval composition remained similar, higher doses of these nutrients in the diet will negatively impact the larval yield. A possible explanation for this observation could be the wellbeing of the larvae in the growth medium. For example, a more lipid-rich environment may change the texture of the substrate. Alternatively, the larval yield may also have been negatively influenced by inhibiting components that were dosed along. In addition, diets 6 and 7 (containing DDGS) had a high concentration of EAA and EFA but a low carbohydrate concentration, which may have resulted in a decreased growth. Based on the results illustrated in Figure 4, diet 15 had the most optimal composition.

**Figure 5:**
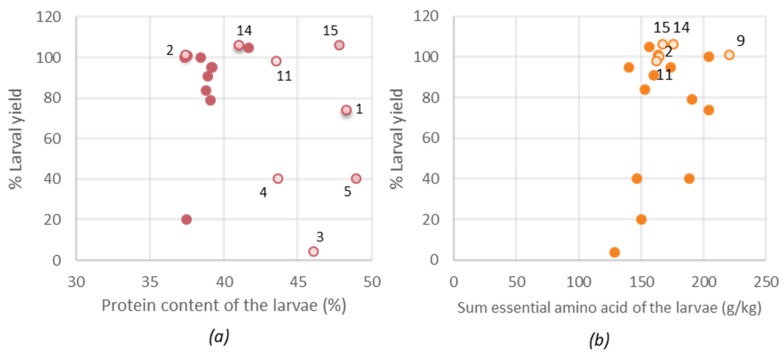
(**a**) The relation between the larval protein and the larval yield, (**b**) The relation between the larval essential amino acid (EAA) content and the larval yield. Hollow bullets were indicated by their diet number. Diet 2 = reference diet.

When the larvae are to be considered as a protein source for food or feed purposes, the optimal nutritional value of the larvae (protein, lipid, EAA and EFA for human consumption) is important, but also the larval yield. Figure 5 illustrates the relationship between the nutritional value and % larval yield. Based on larval protein content, diet 15 (15% rapeseed inclusion) resulted in the most promising diet, referring to the best ratio between larval yield (106%) and protein content (48.%). For diets 1, 3, 4 and 5 a poor yield (< 80% of reference diet) was observed but still a high protein content, potentially referring to enhanced uptake of proteins as a surviving mechanism to compensate for other lacking nutrients. By excluding the diets that resulted in poor yield, the larval lipid content also decreased in variation (from 14–28% to 22–28%). Diet 15 resulted in larvae, next to an increased protein content, also an increased amount of lipids, EAA (>200 g/kg) and a similar amount of EFA compared to the reference diet. Considering the nutritional composition and the relatively larval yield, diet 15 is presumed a good possibility for rearing.

**Figure 6:**
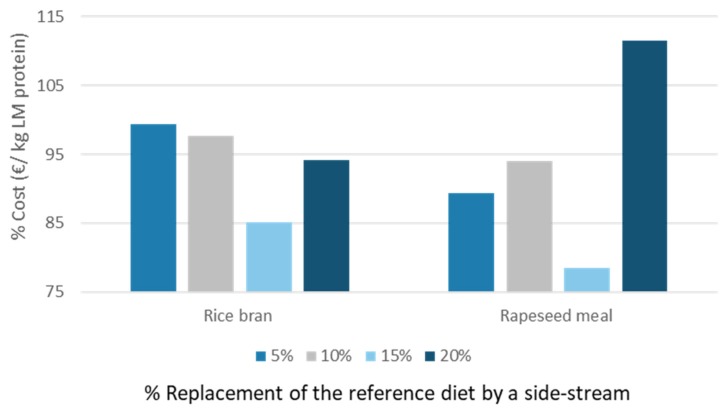
Changes in cost price producing LM larvae proteins with diets 9–16 compared to the reference diet.

Gianotten et al. revealed that the cost of producing LM larvae could be beneficially influenced by including rice bran (up to 20%) or rapeseed meal (up to 10%) [12]. Figure 6 illustrates the results when these calculations were applied to protein level. Diet 15 (15% inclusion of rapeseed) was economically more beneficial than the reference diet (79% of the cost price of diet 2). Next, diet 11 was also found economically beneficial (15% inclusion of rice bran), but larvae reared on this diet have a slightly lower nutritional value in terms of human EAA.

## 4. Conclusions

The study confirmed and complemented published data on the composition of the lesser mealworm. Depending on the diet, the LM larvae had a protein content between 37% and 49%, a lipid content between 14% and 28% (between 22% and 26% for well-performing diets) and a chitin content between 4% and 6%. The most dominant amino acids in the larvae were arginine, alanine, leucine, glutamate, aspartate and tyrosine. The most dominant fatty acids were C16:0, C18:1 and C18:2. The rearing of larvae on underspent side-streams proved to be a good approach for introducing recycled nutrients to the market. The larvae were able to concentrate the proteins and lipids and as a result, also the essential amino acids and fatty acids. In this way, the nutritional value of the side-streams was indirectly raised. With respect to the impact of varying feed ingredients (side-streams) and associated varying concentrations of nutrients, a slight effect on the larval nutrient composition was observed but no direct link to the dietary concentrations could be made. However, the different feed ingredients did influence the larval yield. Within the limitations of the study (type of side-stream, inclusion rates, etc.) a maximum larval yield was achieved with a diet containing 17–22% of proteins and 6–8% of lipids. This finding gives rise to the opportunity to change the diet ingredients or inclusion rates, for example, according to the cost price of the side-streams or the availability, without influencing the larval yield and composition and thus guarantee constant larval biomass in composition. Within the study, diet 15 (wheat middling with 15% inclusion of rapeseed meal + BG) was considered the most interesting diet in terms of larval yield, larval protein content and cost price.

## Figures and Tables

**Table 1:** Overview of all tested diets with the inclusion percentage of side-streams and a summary of the rearing evaluation.

% FM	Wheat Middlings	Rice Bran	Rapeseed Meal	DDGS	Corn Gluten Feed	Moisture Delivery Agent	% Yield *
**Diet1**	100					Carrots	74 ^4^
**Diet2 (ref)**	100					BG	100 ^2^
**Diet3**		100				Carrots	4 ^4^
**Diet4**		100				BG	40 ^4^
**Diet5**			100			Carrots	40 ^4^
**Diet6**				100		Carrots	20 ^4^
**Diet7**				100		BG	79 ^3^
**Diet8**					100	BG	84 ^3^
**Diet9**	95	5				BG	101 ^2^
**Diet10**	90	10				BG	101 ^2^
**Diet11**	85	15				BG	98 ^2^
**Diet12**	80	20				BG	100 ^2^
**Diet13**	95		5			BG	105 ^1^
**Diet14**	90		10			BG	106 ^1^
**Diet15**	85		15			BG	106 ^1^
**Diet16**	80		20			BG	95 ^3^
**Diet17**	50			50		BG	95 ^3^
**Diet18**	50				50	BG	91 ^3^

^1^ excellent yield, ^2^ normal yield, ^3^ tolerable yield, ^4^ bad yield, * calculated and evaluated according to Gianotten et al. [12], BG = brewery grains, FM = fresh matter.

**Table 2:** Results of the simple linear regression analysis between the composition of the diet and the larvae.

Component	R	R^2^	ANOVA Output
Protein content	0.039	0.002	F(1,50) = 0.076; *p* = 0.784
Arginine	0.048	0.002	F(1,45) = 0.102; *p* = 0.751
Hydroxylysine	0.331	0.109	F(1,45) = 5.41; *p* = 0.25
Lysine	0.124	0.015	F(1,45) = 0.704; *p* = 0.406 ^1,2^
Alanine	0.293	0.086	F(1,45) = 4.238; *p* = 0.045
Threonine	0.378	0.143	F(1,45) = 7.494; *p* = 0.009
Glycine	0.021	<0.001	F(1,45) = 0.02; *p* = 0.887
Valine	0.1	0.01	F(1,45) = 0.45; *p* = 0.506
Serine	0.18	0.032	F(1,45) = 1.499; *p* = 0.227
Proline	0.177	0.031	F(1,45) = 1.45; *p* = 0.235
Isoleucine	0.303	0.092	F(1,45) = 4.553; *p* = 0.038
Leucine	0.505	0.255	F(1,45) = 15.424; *p* < 0.001
Methionine	0.031	0.001	F(1,45) = 0.042; *p* = 0.838
Histidine	0.056	0.003	F(1,45) = 0.141; *p* = 0.709
Phenylalanine	0.09	0.008	F(1,45) = 0.366; *p* = 0.548
Glutamate	0.394	0.156	F(1,45) = 8.287; *p* = 0.006
Aspartate	0.033	0.001	F(1,45) = 0.05; *p* = 0.823
Cystine	0.091	0.008	F(1,45) = 0.377; *p* = 0.542 ^1^
Tyrosine	0.255	0.065	F(1,45) = 3.141; *p* = 0.083
Lipid content	0.011	<0.001	F(1,52) = 0.006; *p* = 0.937
C12:0	0.228	0.052	F(1,43) = 2.358; *p* = 0.132
C14:0	0.25	0.062	F(1,52) = 3.458; *p* = 0.069
C14:1	0.189	0.036	F(1,52) = 1.925; *p* = 0.171 ^2^
C16:0	0.195	0.038	F(1,52) = 2.052; *p* = 0.158
C16:1	0.116	0.014	F(1,52) = 0.712; *p* = 0.403
C18:0	0.172	0.029	F(1,52) = 1.576; *p* = 0.215
C18:1	0.38	0.144	F(1,52) = 8.783; *p* = 0.005
C18:2	0.32	0.103	F(1,52) = 5.947; *p* = 0.018
C18:3	0.507	0.257	F(1,52) = 17.985; *p* < 0.001
C18:4	0.125	0.016	F(1,51) = 0.812; *p* = 0.372 ^1,2^
C20:0	0.332	0.11	F(1,52) = 6.45; *p* = 0.014 ^2^
C20:1	0.095	0.009	F(1,52) = 0.47; *p* = 0.496 ^2^
C20:2	0.501	0.251	F(1,45) = 15.057; *p* < 0.001
C20:5	0.136	0.019	F(1,52) = 0.985; *p* = 0.326
C22:0	0.19	0.036	F(1,52) = 1.948; *p* = 0.169 ^2^
C22:1	0.27	0.073	F(1,43) = 3.369; *p* = 0.073 ^1,2^
C20:3	0.015	<0.001	F(1,46) = 0.01; *p* = 0.921
C22:5	0.241	0.058	F(1,52) = 3.199; *p* = 0.08 ^2^
C24:0	0.111	0.012	F(1,52) = 0.645; *p* = 0.425 ^1,2^
C22:6	0.068	0.005	F(1,49) = 0.227; *p* = 0.636 ^1^

^1^ the residuals were not normally distributed, ^2^ homoscedasticity was not valid.

## Abbreviations

AA	Amino acid
BG	Brewery grains
BSFL	Black soldier fly larvae
DDGS	Distillers dried grains with solubles
DM	Dry matter
EAA	Essential amino acids
EFA	Essential fatty acids
FM	Fresh matter
LM	Lesser mealworm
MUFA	mono-unsaturated fatty acids
PUFA	poly-unsaturated fatty acids
R	Pearson correlation coefficient
SD	Standard deviation
SFA	Saturated fatty acids
YM	Yellow mealworm

## Appendix A

**Table A1:** Composition of side-stream ingredients (on dry matter basis, mean ± SD).

	Wheat Middlings	Rice Bran	Rapeseed Meal	DDGS	Corn Gluten Feed	Brewery Grains
**Protein content (%)**	16.2 ± 1.0	14.2 ± 1.8	29.7 ± 0.6	29.2 ± 3.4	16.4 ± 4.6	20.7 ± 0.4
**Amino acids (g/kg)**						
**Arginine^E^**	46.2 ± 5.3	70.4 ± 8.8	73.9 ± 11.1	108.7 ± 34.5	39.0 ± 10.8	29.9 ± 2.8
**Alanine**	7.5 ± 0.5	6.9 ± 1.1	14.1 ± 0.8	19.2 ± 1.8	15.7 ± 4.6	12.0 ± 0.3
**Aspartate**	12.2 ± 0.7	11.2 ± 1.2	23.9 ± 2.4	17.1 ± 0.5	9.7 ± 2.5	15.1 ± 0.2
**Cystine**	1.7 ± 0.1	1.4 ± 0.1	4.7 ± 0.2	3.1 ± 0.3	1.1 ± 0.4	3.2 ± 0.1
**Glutamate**	34.7 ± 1.6	16.1 ± 2.4	59.8 ± 6.8	45.9 ± 0.7	32.5 ± 9.9	52.3 ± 2.1
**Glycine**	7.9 ± 0.9	6.2 ± 0.7	16.6 ± 0.8	11.0 ± 0.7	7.6 ± 2.1	9.5 ± 0.2
**Histidine ^E^**	2.7 ± 0.1	2.1 ± 0.5	7.7 ± 0.4	5.5 ± 1.6	5.9 ± 1.6	5.0 ± 0.3
**Hydroxylysine**	0.04 ± 0.01	0.02 ± 0.02	0.06 ± 0.10	0.03 ± 0.06	0.0 ± 0.0	0.01 ± 0.00
**Isoleucine ^E^**	4.7 ± 0.2	3.4 ± 0.5	12.4 ± 1.4	8.5 ± 0.3	5.4 ± 1.5	9.3 ± 0.0
**Leucine ^E^**	11.5 ± 0.8	8.2 ± 0.9	23.5 ± 1.5	31.3 ± 2.7	18.0 ± 5.0	20.2 ± 0.1
**Lysine ^E^**	5.6 ± 0.7	4.8 ± 0.7	15.3 ± 1.4	6.8 ± 1.1	6.4 ± 1.7	2.7 ± 0.1
**Methionine ^E^**	3.3 ± 0.4	2.4 ± 0.4	5.1 ± 1.2	3.7 ± 1.1	0.3 ± 0.1	2.9 ± 0.3
**Phenylalanine^E^**	6.9 ± 0.3	4.7 ± 0.2	13.1 ± 0.6	11.7 ± 1.9	7.0 ± 2.0	14.2 ± 0.2
**Proline**	9.9 ± 1.3	5.4 ± 0.5	16.9 ± 0.5	18.3 ± 0.8	15.5 ± 4.0	23.9 ± 0.4
**Serine**	6.7 ± 0.7	4.8 ± 0.8	7.6 ± 6.6	10.7 ± 0.2	7.5 ± 2.3	9.4 ± 0.1
**Threonine ^E^**	5.6 ± 0.5	4.9 ± 0.9	14.6 ± 1.7	9.5 ± 0.2	6.8 ± 1.8	9.1 ± 0.1
**Tyrosine**	3.7 ± 0.4	2.8 ± 0.3	8.5 ± 0.1	9.2 ± 0.7	4.4 ± 1.2	7.6 ± 0.1
**Valine ^E^**	7.8 ± 0.4	6.7 ± 1.7	19.5 ± 1.3	15.9 ± 1.5	8.0 ± 2.1	13.7 ± 0.3
**Sum EAA (g/kg)**	98.7 ± 6.6	107.5 ± 14.0	188.3 ± 2.2	201.7 ± 44.9	96.7 ± 26.6	107.1 ± 3.8
**Lipid content (%)**	4.3 ± 0.1	18.7 ± 0.5	1.7 ± 0.1	14.2 ± 2.4	1.6 ± 0.1	9.8 ± 0.1
**Fatty acids (mg/kg)**						
**C12:0**	755.7 ± 11.8	nd	6.0 ± 5.7	46.9 ± 40.1	10.2 ± 3.2	15.9 ± 4.3
**C14:0**	306.6 ± 3.3	721.6 ± 30.4	24.4 ± 18.3	42.5 ± 39.2	9.1 ± 3.3	248.9 ± 47.9
**C14:1**	12.3 ± 2.0	189.2 ± 30.7	4.0 ± 2.0	8.0 ± 8.6	7.8 ± 7.6	8.6 ± 1.6
**C16:0**	5.213.5 ± 140.2	22.468.5 ± 646.6	677.7 ± 465.5	13.473.1 ± 2.472.6	1.708.9 ± 755.5	12.779.8 ± 4.134.3
**C16:1**	184.5 ± 75.6	1.049.0 ± 99.9	230.8 ± 137.2	365.6 ± 236.2	17.5 ± 7.2	1.377.4 ± 499.2
**C18:0**	290.9 ± 6.6	1.435.9 ± 64.2	70.2 ± 64.0	1.025.7 ± 218.6	242.8 ± 109.4	639.7 ± 168.3
**C18:1**	6.001.7 ± 166.7	62.398.7 ± 521.2	3.257.7 ± 2.677.1	32.120.8 ± 5.945.6	2.965.6 ± 1.220.6	5.818.5 ± 1.191.5
**C18:2^E^**	17907.9 ± 605.7	53336.2 ± 279.6	1829.8 ± 1060.9	59517.4 ± 11442.9	6.454.9 ± 2.526.7	27469.1 ± 4949.9
**C18:3 ^E^**	1765.3 ± 65.6	1764.8 ± 14.7	348.8 ± 247.5	1456.1 ± 260.6	373.6 ± 143.9	3020.5 ± 539.6
**C18:4**	nd	48.4 ± 1.2	9.0 ± 15.6	41.5 ± 13.2	6.7 ± 1.6	nd
**C20:0**	54.2 ± 5.0	1098.4 ± 119.8	155.9 ± 130.8	392.9 ± 61.5	67.3 ± 29.3	127.7 ± 28.4
**C20:1**	270.6 ± 10.0	1409.2 ± 298.0	81.5 ± 132.6	454.5 ± 94.0	48.0 ± 23.8	451.0 ± 105.4
**C20:2**	87.8 ± 13.7	285.8 ± 68.1	33.9 ± 17.5	20.4 ± 18.9	47.0 ± 3.9	nd
**C20:5 ^E^**	20.3 ± 17.6	nd	5.9 ± 10.1	27.1 ± 23.8	5.8 ± 5.6	93.2 ± 20.2
**C22:0**	223.1 ± 22.7	1731.5 ± 406.0	125.5 ± 93.4	334.4 ± 50.0	19.3 ± 5.9	334.8 ± 87.3
**C22:1**	nd	nd	nd	nd	nd	73.2 ± 14.0
**C20:3**	53.8 ± 22.8	281.7 ± 67.3	12.7 ± 9.4	126.1 ± 61.8	344.8 ± 100.8	nd
**C22:5 ^E^**	3.5 ± 6.1	nd	2.0 ± 3.5	nd	nd	334.4 ± 54.6
**C24:0**	60.0 ± 6.2	2231.6 ± 581.8	126.0 ± 139.5	217.9 ± 40.0	38.9 ± 17.8	553.4 ± 130.8
**C22:6 ^E^**	18.5 ± 17.0	207.0 ± 292.7	80.5 ± 125.0	22.8 ± 20.0	nd	109.5 ± 55.2
**Sum EFA (g/kg)**	19.7 ± 0.7	55.3 ± 0.6	3.04 ± 0.0	61.0 ± 11.7	6.8 ± 2.7	31.0 ± 5.6
**Sum SFA**	6.9 ± 0.2	29.7 ± 1.8	1.2 ± 0.9	15.5 ± 2.9	2.1 ± 0.9	14.7 ± 4.6
**Sum MUFA**	6.5 ± 0.3	65.0 ± 0.9	3.6 ± 2.9	32.9 ± 6.3	3.0 ± 1.3	7.7 ± 1.8
**Sum PUFA**	19.9 ± 0.7	55.9 ± 0.7	2.3 ± 1.5	61.2 ± 11.8	6.8 ± 2.7	31.4 ± 5.7
**% Starch ***	21.8	32.7	0	4.8	15.3	3.7
**% Sugar ***	6.3	4.1	8.3	1.5	2.4	2.4
**Total ***	28.1	36.8	8.3	6.3	17.7	6.1

EAA: essential amino acids for insects, EFA: essential fatty acids, ^E^ = essential, SFE: saturated fatty acids, MUFA: monounsaturated fatty acid, PUFA: polyunsaturated fatty acids, * according to the CVB (Centraal Veevoederbureau) table (2018) [13], nd = not detected.

**Table A2:** Protein content (%), amino acid profile (g/kg) and sum of essential amino acids (g/kg) of the diets on dry matter basis (mean ± SD). ^E^ essential amino acids for insects, EAA: essential amino acids, ** ANOVA analysis followed by Tukey post-hoc (letters in superscript), others were analyzed by Kruskal–Wallis.

	Diet 1	Diet 2	Diet 3	Diet 4	Diet 5	Diet 6	Diet 7	Diet 8	Diet 9	Diet 10	Diet 11	Diet 12	Diet 13	Diet 14	Diet 15	Diet 16	Diet 17	Diet 18
**Protein content (%)**	15.5 ± 1.0^ab^	17.2 ± 0.6 ^abc^	14.2 ± 1.8 ^a^	17.9 ± 0.8 ^abcd^	29.2 ± 0.6 ^f^	29.2 ± 3.4 ^ef^	26.4 ± 2.2 ^ef^	17.9 ± 3.2 ^abcd^	17.1 ± 0.5 ^abc^	17.1 ± 0.5 ^abc^	17.1 ± 0.5 ^abc^	17.0 ± 0.4 ^abc^	17.7 ± 0.5 ^abcd^	18.2 ± 0.5 ^abcd^	18.6 ± 0.4 ^bcd^	19.1 ± 0.4 ^cd^	21.8 ± 1.3 ^de^	17.6 ± 1.4 ^abcd^
**Arginine**	46.2 ± 5.3 ^ab^	41.0 ± 2.9 ^ab^	70.4 ± 8.8 ^cd^	47.4 ± 4.5 ^abc^	73.9 ± 11.1 ^cd^	108.7 ± 34.5 ^d^	82.6 ± 22.4 ^cd^	35.9 ± 7.80 ^ab^	41.8 ± 2.9 ^ab^	42.6 ± 3.0 ^ab^	43.4 ± 3.0 ^ab^	44.2 ± 3.1 ^ab^	41.6 ± 2.5 ^ab^	42.5 ± 2.3 ^ab^	43.5 ± 2.1 ^ab^	44.4 ± 2.0 ^ab^	61.7 ± 12.6 ^bcd^	38.2 ± 2.5 ^a^
**Alanine**	7.5 ± 0.5 ^ab^	8.9 ± 0.3 ^abcd^	6.9 ± 1.1^a^	9.8 ± 0.5 ^def^	14.1 ± 0.8 ^hi^	19.2 ± 1.8 ^j^	16.8 ± 1.1 ^ij^	14.5 ± 3.1 ^hij^	9.0 ± 0.2 ^abc^	8.9 ± 0.2 ^abc^	8.9 ± 0.2 ^abc^	8.9 ± 0.2 ^abc^	9.3 ± 0.2 ^bcde^	9.5 ± 0.2 ^cdef^	9.7 ± 0.2 ^def^	9.9 ± 0.1 ^def^	12.9 ± 0.7 ^ghi^	11.7 ± 1.5 ^fghi^
**Aspartate**	12.2 ± 0.7 ^abc^	13.1 ± 0.5 ^bcd^	11.2 ± 1.2 ^a^	13.4 ± 0.4 ^bcd^	23.9 ± 2.4 ^h^	17.1 ± 0.5 ^g^	16.4 ± 0.4 ^fg^	11.5 ± 1.7 ^ab^	13.1 ± 0.4 ^bcd^	13.1 ± 0.4 ^bcd^	13.0 ± 0.3 ^abcd^	13.0 ± 0.3 ^abcd^	13.6 ± 0.4 ^bcd^	14.0 ± 0.4 ^cde^	14.3 ± 0.3 ^def^	14.7 ± 0.3 ^ef^	14.8 ± 0.4 ^ef^	12.4 ± 0.8 ^abc^
**Cystine**	1.7 ± 0.1 ^ab^	2.2 ± 0.1^cdef^	1.4 ± 0.1^a^	2.4 ± 0.1^fghi^	4.7 ± 0.2 ^l^	3.1 ± 0.3 ^jkl^	3.1 ± 0.2 ^kl^	1.8 ± 0.3 ^abc^	2.2 ± 0.1 ^cde^	2.2 ± 0.1 ^cde^	2.2 ± 0.1 ^cde^	2.2 ± 0.1 ^bcd^	2.3 ± 0.1 ^defg^	2.4 ± 0.1 ^efgh^	2.5 ± 0.1 ^ghi^	2.6 ± 0.1 ^hij^	2.7 ± 0.1 ^ijk^	2.0 ± 0.2 ^abcd^
**Glutamate**	34.7 ± 1.6 ^b^	40.3 ± 1.4 ^bcdef^	16.1 ± 2.4 ^a^	36.7 ± 0.3 ^bc^	59.8 ± 6.8 ^i^	45.9 ± 0.7 ^g^	48.0 ± 1.1 ^h^	39.1 ± 6.9 ^bcdef^	39.7 ± 1.3 ^bcde^	39.1 ± 1.2 ^bcde^	38.5 ± 1.0 ^bcd^	37.8 ± 0.9 ^bcd^	41.5 ± 1.2 ^bcdef^	42.3 ± 1.1 ^cdef^	43.1 ± 1.0 ^defg^	44.0 ± 1.0 ^efg^	44.3 ± 1.0 ^fg^	40.1 ± 3.2 ^bcdef^
**Glycine ****	7.9 ± 0.9 ^ab^	8.4 ± 0.6 ^b^	6.2 ± 0.7^a^	8.0 ± 0.4 ^ab^	16.6 ± 0.8 ^e^	11.0 ± 0.7 ^d^	10.5 ± 0.4 ^cd^	8.2 ± 1.4 ^b^	8.4 ± 0.5 ^b^	8.3 ± 0.5 ^b^	8.2 ± 0.4 ^b^	8.2 ± 0.4 ^b^	8.7 ± 0.5 ^bc^	9.0 ± 0.5 ^bc^	9.3 ± 0.5 ^bcd^	9.6 ± 0.5 ^bcd^	9.5 ± 0.4 ^bcd^	8.4 ± 0.5 ^b^
**Histidine ^E^**	2.7 ± 0.1 ^b^	3.4 ± 0.1 ^bcd^	2.1 ± 0.5 ^a^	3.8 ± 0.4 ^cdef^	7.7 ± 0.4 ^g^	5.4 ± 1.6 ^efg^	5.3 ± 0.9 ^efg^	5.6 ± 1.1 ^fg^	3.4 ± 0.1 ^bc^	3.4 ± 0.1 ^bc^	3.4 ± 0.1 ^bc^	3.3 ± 0.1 ^bc^	3.6 ± 0.1 ^cde^	3.8 ± 0.1 ^cdef^	4.0 ± 0.1^defg^	4.1 ± 0.1 ^efg^	4.4 ± 0.5 ^efg^	4.5 ± 0.5 ^efg^
**Isoleucine^E^**	4.7 ± 0.2 ^b^	6.2 ± 0.2 ^bcd^	3.4 ± 0.5 ^a^	6.8 ± 0.2 ^cd^	12.4 ± 1.4 ^g^	8.5 ± 0.3 ^f^	8.8 ± 0.2 ^f^	6.7 ± 1.0 ^bcd^	6.1 ± 0.2 ^bc^	6.1 ± 0.1 ^bc^	6.0 ± 0.1 ^bc^	6.0 ± 0.1 ^bc^	6.5 ± 0.1 ^bcd^	6.8 ± 0.1 ^bcd^	7.0 ± 0.1 ^cde^	7.3 ± 0.2 ^de^	7.5 ± 0.1 ^e^	6.5 ± 0.6 ^bcd^
**Leucine ^E^**	11.5 ± 0.8 ^b^	14.3 ± 0.6 ^bcdef^	8.2 ± 0.9 ^a^	15.0 ± 0.4 ^cdefg^	23.5 ± 1.5 ^h^	31.3 ± 2.7 ^i^	27.6 ± 1.8 ^i^	18.7 ± 3.4 ^fgh^	14.2 ± 0.5 ^bcde^	14.1 ± 0.4 ^bcd^	14.0 ± 0.4 ^bc^	13.8 ± 0.3 ^bc^	14.8 ± 0.5 ^cdefg^	15.2 ± 0.4 ^defg^	15.6 ± 0.3 ^efg^	16.0 ± 0.2 ^fg^	21.0 ± 1.1 ^gh^	16.6 ± 1.8 ^defg^
**Lysine ^E^**	5.6 ± 0.7 ^abc^	4.7 ± 0.5 ^ab^	4.8 ± 0.7 ^abc^	3.6 ± 0.2 ^a^	15.3 ± 1.4 ^c^	6.8 ± 1.1 ^bc^	5.5 ± 0.8 ^abc^	5.2 ± 1.1 ^abc^	4.7 ± 0.5 ^ab^	4.6 ± 0.5 ^ab^	4.6 ± 0.5 ^ab^	4.6 ± 0.4 ^ab^	5.0 ± 0.5 ^abc^	5.3 ± 0.5 ^abc^	5.6 ± 0.5 ^abc^	5.9 ± 0.5 ^bc^	5.1 ± 0.5 ^abc^	4.9 ± 0.4 ^abc^
**Methionine ^E^**	3.3 ± 0.4 ^cde^	3.2 ± 0.2 ^cde^	2.4 ± 0.4 ^abc^	2.6 ± 0.3 ^bcd^	5.1 ± 1.3 ^e^	3.7 ± 1.1 ^cde^	3.4 ± 0.8 ^cde^	1.1 ± 0.1^a^	3.1 ± 0.2 ^cde^	3.1 ± 0.1 ^cde^	3.1 ± 0.1 ^cde^	3.0 ± 0.1 ^cde^	3.2 ± 0.1 ^cde^	3.3 ± 0.1 ^cde^	3.3 ± 0.1 ^de^	3.4 ± 0.1 ^de^	3.3 ± 0.3 ^cde^	2.2 ± 0.0 ^ab^
**Phenylalanine ^E^**	6.9 ± 0.3 ^b^	9.3 ± 0.2 ^bcde^	4.7 ± 0.2 ^a^	10.1 ± 0.1^defgh^	13.1 ± 0.6 ^h^	11.7 ± 1.9 ^efgh^	12.6 ± 1.3 ^gh^	9.4 ± 1.4 ^bcdefg^	9.2 ± 0.2 ^bcd^	9.1 ± 0.2 ^bcd^	9.1 ± 0.1 ^bc^	9.0 ± 0.1 ^bc^	9.6 ± 0.2 ^bcdef^	9.8 ± 0.1 ^bcdefg^	10.0 ± 0.1 ^cdefg^	10.2 ± 0.1 ^efgh^	11.0 ± 0.7 ^fgh^	9.5 ± 0.7 ^bcdef^
**Proline**	9.9 ± 1.3 ^b^	14.4 ± 0.9 ^bcdef^	5.4 ± 0.5 ^a^	15.9 ± 0.3 ^defghi^	16.9 ± 0.5 ^fghi^	18.3 ± 0.8 ^hi^	20.2 ± 0.7 ^i^	18.3 ± 2.8 ^fghi^	14.3 ± 0.8 ^bcdef^	14.2 ± 0.8 ^bcde^	14.0 ± 0.7 ^bcd^	13.9 ± 0.6 ^bc^	14.9 ± 0.8 ^bcdefg^	15.1 ± 0.8 ^bcdefgh^	15.4 ± 0.7 ^cde^	15.6 ± 0.7 ^cdefgh^	17.4 ± 0.5 ^ghi^	16.6 ± 1.3 ^efghi^
**Serine**	6.7 ± 0.7 ^ab^	7.6 ± 0.4 ^ab^	4.8 ± 0.8 ^a^	7.4 ± 0.4 ^ab^	7.6 ± 6.6 ^ab^	10.7 ± 0.2 ^b^	10.3 ± 0.1 ^ab^	8.1 ± 1.5 ^ab^	7.5 ± 0.4 ^ab^	7.5 ± 0.3 ^ab^	7.4 ± 0.3 ^ab^	7.3 ± 0.3 ^ab^	7.7 ± 0.3 ^ab^	7.7 ± 0.4 ^ab^	7.7 ± 0.6 ^ab^	7.8 ± 0.8 ^ab^	8.9 ± 0.2 ^ab^	7.9 ± 0.7 ^ab^
**Threonine ^E^**	5.6 ± 0.5 ^ab^	6.8 ± 0.3 ^bcd^	4.9 ± 1.0 ^a^	7.3 ± 0.4 ^bcd^	14.6 ± 1.7 ^g^	9.5 ± 0.2 ^fg^	9.4 ± 0.1 ^efg^	7.6 ± 1.2 ^bcde^	6.7 ± 0.3 ^bc^	6.7 ± 0.3 ^bc^	6.7 ± 0.3 ^bc^	6.7 ± 0.3 ^bc^	7.1 ± 0.3 ^bcd^	7.4 ± 0.3 ^bde^	7.7 ± 0.2 ^cde^	8.0 ± 0.2^cde^	8.1 ± 0.2 ^def^	7.2 ± 0.5 ^bcd^
**Tyrosine**	3.7 ± 0.4 ^b^	4.9 ± 0.3 ^bcd^	2.8 ± 0.3 ^a^	5.5 ± 0.2 ^cd^	8.5 ± 0.1 ^e^	9.2 ± 0.7 ^e^	8.7 ± 0.5 ^e^	5.4 ± 0.8 ^bcd^	4.9 ± 0.3 ^bcd^	4.9 ± 0.2 ^bc^	4.8 ± 0.2 ^bc^	4.8 ± 0.2 ^bc^	5.1 ± 0.3 ^bcd^	5.3 ± 0.2 ^bcd^	5.5 ± 0.2 ^bcd^	5.6 ± 0.2 ^cd^	6.8 ± 0.4 ^de^	5.2 ± 0.4 ^bcd^
**Valine ^E^**	7.8 ± 0.4 ^ab^	9.7 ± 0.4 ^abcd^	6.7 ± 1.7 ^a^	10.7 ± 0.8 ^bcde^	19.5 ± 1.3 ^g^	15.9 ± 1.5 ^fg^	15.2 ± 0.9 ^fg^	9.9 ± 1.5 ^abcde^	9.7 ± 0.4 ^abcd^	9.7 ± 0.4 ^abcd^	9.6 ± 0.4 ^abc^	9.6 ± 0.5 ^abc^	10.2 ± 0.4 ^abcde^	10.6 ± 0.4 ^bcde^	11.0 ± 0.4 ^cde^	11.4 ± 0.5 ^de^	12.5 ± 0.3 ^ef^	9.9 ± 0.8 ^abcd^
**Sum of EAA (g/kg)**	94.40 ± 6.31^a^	98.47 ± 3.27 ^ab^	107.52 ± 13.99 ^ab^	107.27 ± 7.01 ^ab^	185.16 ± 2.32 ^c^	201.65 ± 37.88 ^c^	170.23 ± 24.27 ^c^	100.15 ± 18.39 ^ab^	98.94 ± 3.24 ^ab^	99.38 ± 3.30 ^ab^	99.83 ± 3.43 ^ab^	100.27 ± 3.62 ^ab^	101.70 ± 2.89 ^ab^	104.70 ± 2.67 ^ab^	107.70 ± 2.45 ^ab^	110.70 ± 2.23 ^ab^	134.42 ± 13.72 ^bc^	99.53 ± 7.57 ^ab^

**Table A3:** Lipid content (%), fatty acid profile (mg/kg) and sum of essential fatty acids (g/kg) of the diets on dry matter basis (mean ± SD), ^E^ essential fatty acids, * analysis performed in duplicate, EFA: essential fatty acids, SFE: saturated fatty acids, MUFA: monounsaturated fatty acid, PUFA: polyunsaturated fatty acids, nd: not detected, ** ANOVA analysis followed by Tukey post-hoc (letters in superscript), others were analyzed by Kruskal–Wallis (^ indicates if no significant differences were observed).

	Diet 1	Diet 2	Diet 3 *	Diet 4	Diet 5	Diet 6	Diet 7	Diet 8	Diet 9	Diet 10	Diet 11	Diet 12	Diet 13	Diet 14	Diet 15	Diet 16	Diet 17	Diet 18
**Lipid content (%)**	4.3 ± 0.1 ^ab^	6.1 ± 0.1 ^ef^	18.7 ± 0.5 ^k^	13.7 ± 0.3 ^jk^	1.7 ± 0.1 ^a^	14.2 ± 2.4 ^jk^	12.8 ± 1.6 ^jk^	4.3 ± 0.1 ^ab^	6.6 ± 0.1 ^f^	7.1 ± 0.1 ^g^	7.6 ± 0.1 ^h^	8.1 ± 0.1 ^i^	6.1 ± 0.1 ^ef^	6.0 ± 0.1 ^def^	5.9 ± 0.1 ^cde^	5.8 ± 0.1 ^cd^	9.5 ± 0.8 ^j^	5.3 ± 0.0 ^bc^
**C12:0**	755.7 ± 11.8 ^k^	518.3 ± 8.9 ^j^	Nd	14.4 ± 11.5 ^ab^	3.1 ± 2.6 ^a^	46.9 ± 40.1 ^ab^	36.6 ± 26.4 ^ab^	12.1 ± 3.4 ^ab^	491.5 ± 8.7 ^ij^	466.3 ± 8.6 ^ghi^	441.2 ± 8.6 ^efg^	416.0 ± 8.7 ^cde^	480.2 ± 8.3 ^hij^	455.3 ± 7.8 ^fgh^	430.4 ± 7.4^def^	405.6 ± 6.9 ^cd^	273.2 ± 15.9 ^c^	256.1 ± 5.3 ^b^
**C14:0 ****	306.6 ± 3.3 ^efg^	288.1 ± 17.4 ^efg^	721.6 ± 30.4 ^i^	451.2 ± 18.1 ^h^	35.0 ± 0.5 ^a^	42.5 ± 39.2 ^a^	111.1 ± 42.1 ^b^	89.3 ± 15.8 ^ab^	301.9 ± 16.6 ^efg^	315.9 ± 15.8 ^efg^	329.8 ± 15.0 ^fg^	343.8 ± 14.2 ^g^	278.1 ± 18.0 ^ef^	269.1 ± 17.9 ^ef^	260.1 ± 17.8 ^de^	251.1 ± 17.7 ^cde^	199.3 ± 30.0 ^cd^	189.3 ± 17.3 ^c^
**C14:1**	12.3 ± 2.0 ^a^	11.1 ± 1.7 ^a^	189.2 ± 30.7 ^d^	75.0 ± 21.5 ^c^	5.1 ± 0.9 ^a^	8.0 ± 8.6 ^a^	8.2 ± 5.9 ^a^	8.1 ± 4.9 ^a^	16.2 ± 2.7 ^ab^	21.3 ± 4.2 ^abc^	26.4 ± 5.8 ^bc^	31.5 ± 7.4 ^bc^	10.8 ± 1.6 ^a^	10.6 ± 1.5 ^a^	10.3 ± 1.4 ^a^	10.1 ± 1.3 ^a^	9.6 ± 2.7 ^a^	9.6 ± 1.8 ^a^
**C16:0****	5213.5 ± 140.2 ^ab^	7641.9 ± 1422.1 ^bc^	22468.5 ± 646.6 ^f^	16891.5 ± 2174.4 ^e^	946.4 ± 9.1 ^a^	13473.1 ± 2472.6 ^de^	13242.7 ± 2540.8^de^	5412.3 ± 1416.0 ^ab^	8237.5 ± 1411.5 ^bc^	8816.9 ± 1392.3 ^bcd^	9396.3 ± 1373.3 ^bcd^	9975.8 ± 1354.3 ^bcd^	7635.5 ± 1488.2 ^bc^	7494.4 ± 1483.3 ^bc^	7353.3 ± 1478.4 ^bc^	7212.2 ± 1473.4 ^bc^	10485.3 ± 1910.9 ^cd^	6680.4 ± 1466.4 ^bc^
**C16:1 ****	184.5 ± 75.6 ^a^	567.3 ± 168.8 ^ab^	1049.0 ± 99.9 ^bc^	1256.5 ± 238.1 ^c^	308.4 ± 27.5 ^a^	365.6 ± 236.2 ^a^	701.8 ± 323.5 ^abc^	472.4 ± 163.8 ^a^	600.8 ± 165.4 ^ab^	631.6 ± 161.0 ^ab^	662.5 ± 156.7 ^ab^	693.3 ± 152.3 ^ab^	592.7 ± 175.7 ^ab^	596.8 ± 174.5 ^ab^	600.8 ± 173.3 ^ab^	604.9 ± 172.2 ^ab^	641.4 ± 246.0 ^ab^	541.9 ± 172.7 ^ab^
**C18:0**	290.9 ± 6.6 ^ab^	402.8 ± 56.7 ^abc^	1435.9 ± 64.2 ^g^	951.0 ± 88.8 ^fg^	101.9 ± 32.6 ^a^	1025.7 ± 218.6 ^fg^	897.4 ± 187.3 ^efg^	375.6 ± 95.6 ^abc^	439.8 ± 55.5 ^abcd^	476.1 ± 54.2 ^abcd^	512.4 ± 53.2 ^bcde^	548.7 ± 52.6 ^cde^	402.8 ± 58.4 ^abc^	396.5 ± 57.2 ^abc^	390.3 ± 56.0 ^abc^	384.0 ± 54.8 ^abc^	652.1 ± 119.4^def^	395.6 ± 70.9 ^abc^
**C18:1**	6001.7 ± 166.7 ^bc^	5942.9 ± 489.9 ^bc^	62398.7 ± 521.2 ^h^	28270.1 ± 3420.7 ^gh^	4803.2 ± 29.5 ^ab^	32120.8 ± 5945.6 ^gh^	23380.1 ± 4164.3 ^fg^	3919.9 ± 860.4 ^a^	7701.8 ± 588.7 ^c^	9461.2 ± 763.1 ^d^	11220.7 ± 974.5 ^de^	12980.2 ± 1203.5 ^def^	5900.0 ± 503.9 ^bc^	5860.4 ± 499.6 ^bc^	5820.7 ± 495.2 ^bc^	5781.1 ± 490.9 ^bc^	14660.4 ± 2239.9 ^ef^	4944.8 ± 560.3 ^ab^
**C18:2 ^E^**	17907.9 ± 605.7 ^bc^	20976.6 ± 1988.7^bcde^	53336.2 ± 279.6 ^fgh^	34361.0 ± 8063.6 ^fgh^	2441.8 ± 41.6 ^a^	59517.4 ± 11442.9 ^h^	48867.3 ± 8413.7 ^gh^	13484.5 ± 2332.5 ^b^	21862.3 ± 2129.6 ^cdef^	22727.8 ± 2393.8 ^cdef^	23593.4 ± 2752.8 ^cdefg^	24459.0 ± 3174.6 ^defgh^	20635.4 ± 2047.9 ^bcde^	20124.0 ± 2029.7 ^bcde^	19612.7 ± 2011.5 ^bcde^	19101.3 ± 1993.3 ^bcde^	34976.2 ± 4877.0 ^efgh^	17462.8 ± 2035.7 ^bcd^
**C18:3 ^E^**	1765.3 ± 65.6 ^abcd^	2168.2 ± 216.8 ^d^	1764.8 ± 14.7 ^abcd^	2279.9 ± 484.1 ^d^	490.4 ± 33.4 ^a^	1456.1 ± 260.6 ^abc^	1976.0 ± 297.0 ^bcd^	1259.1 ± 204.1 ^ab^	2155.1 ± 221.0 ^d^	2139.3 ± 227.3 ^d^	2123.5 ± 236.5 ^d^	2107.8 ± 248.2 ^d^	2148.4 ± 224.2 ^d^	2106.2 ± 223.1 ^d^	2064.0 ± 222.1 ^d^	2021.9 ± 221.1 ^cd^	2079.2 ± 251.1 ^d^	1743.5 ± 211.4 ^abcd^
**C18:4**	nd	nd	48.4 ± 1.2 ^e^	207.7 ± 323.7 ^de^	13.5 ± 13.5 ^abcde^	41.5 ± 13.2 ^de^	27.7 ± 8.8 ^cde^	4.4 ± 1.1 ^abcde^	16.3 ± 25.5 ^abcde^	32.7 ± 50.9 ^abcde^	49.0 ± 76.4 ^bcde^	65.4 ± 101.8 ^bcde^	0.4 ± 0.4 ^a^	0.9 ± 0.9 ^ab^	1.3 ± 1.3 ^abc^	1.8 ± 1.8 ^abcd^	13.8 ± 4.4 ^abcde^	2.2 ± 0.5 ^abcde^
**C20:0**	54.2 ± 5.0 ^a^	77.8 ± 11.4 ^ab^	1098.4 ± 119.8 ^h^	593.4 ± 80.7 ^g^	231.3 ± 3.9 ^de^	392.9 ± 61.5 ^f^	304.7 ± 47.8 ^ef^	87.5 ± 18.5 ^ab^	117.1 ± 6.7 ^b^	156.2 ± 8.1 ^c^	195.3 ± 13.9 ^cd^	234.4 ± 20.7 ^de^	85.0 ± 11.8 ^ab^	90.8 ± 11.8 ^ab^	96.7 ± 11.8 ^ab^	102.5 ± 11.8 ^ab^	191.7 ± 29.2 ^cd^	83.9 ± 10.6 ^ab^
**C20:1**	270.6 ± 10.0 ^abc^	328.5 ± 30.5 ^bcdef^	1409.2 ± 298.0 ^i^	1155.0 ± 515.1 ^hi^	117.3 ± 117.3 ^a^	454.5 ± 94.0 ^defgh^	453.4 ± 83.5 ^defgh^	182.8 ± 38.3 ^a^	390.4 ± 45.6 ^cdefgh^	451.8 ± 80.8 ^efgh^	513.3 ± 119.5 ^fgh^	574.8 ± 159.1 ^ghi^	326.6 ± 36.2 ^bcdefg^	321.6 ± 40.0 ^bcde^	316.5 ± 43.9 ^bcd^	311.4 ± 47.9 ^bcd^	392.0 ± 55.7 ^ab^	260.1 ± 34.7 ^cdefgh^
**C20:2**	87.8 ± 13.7^defgh^	74.7 ± 8.8 ^defg^	285.8 ± 68.1 ^h^	149.8 ± 26.1 ^gh^	43.9 ± 1.6 ^bc^	20.4 ± 18.9 ^a^	29.2 ± 13.2 ^ab^	15.7 ± 1.3 ^a^	87.8 ± 19.5 ^defgh^	100.9 ± 30.3 ^efgh^	114.0 ± 41.0 ^efgh^	127.1 ± 51.8 ^fgh^	72.6 ± 8.1 ^defg^	71.1 ± 7.7 ^def^	69.7 ± 7.2 ^de^	68.2 ± 6.7 ^de^	51.7 ± 2.4 ^cd^	45.0 ± 4.1 ^abc^
**C20:5 ^E^ ****	20.3 ± 17.6^ab^	43.7 ± 14.9 ^b^	nd	53.1 ± 11.5 ^b^	nd	27.1 ± 23.8 ^ab^	49.0 ± 19.9 ^b^	35.0 ± 4.6 ^ab^	43.1 ± 14.4 ^b^	42.4 ± 13.8 ^ab^	41.8 ± 13.3 ^ab^	41.1 ± 12.8 ^ab^	44.3 ± 14.3 ^b^	43.6 ± 13.8 ^b^	42.9 ± 13.3 ^b^	42.3 ± 12.8 ^ab^	46.8 ± 17.4 ^b^	40.7 ± 8.3 ^ab^
**C22:0**	223.1 ± 22.7 ^abcd^	259.0 ± 24.6 ^cdef^	1731.5 ± 406.0 ^k^	1189.3 ± 435.3 ^jk^	176.9 ± 28.5 ^ab^	334.4 ± 50.0 ^efgh^	334.6 ± 60.6 ^defgh^	124.9 ± 29.4 ^a^	330.2 ± 19.6 ^fgh^	401.2 ± 52.8 ^ghi^	472.1 ± 88.5 ^hij^	543.1 ± 124.5 ^ij^	259.4 ± 25.1 ^cdefg^	257.9 ± 24.4 ^cde^	256.4 ± 23.7 ^cde^	254.8 ± 23.0 ^bcde^	297.4 ± 42.6 ^cdefgh^	195.0 ± 27.8 ^abc^
**C22:1****	nd	23.5 ± 4.5 ^a^	nd	41.7 ± 8.0 ^b^	nd	nd	24.3 ± 4.7 ^a^	24.5 ± 4.7 ^a^	23.7 ± 4.5 ^a^	23.7 ± 4.5 ^a^	23.7 ± 4.5 ^a^	23.7 ± 4.5 ^a^	24.8 ± 4.8 ^a^	24.8 ± 4.8 ^a^	24.8 ± 4.8 ^a^	24.8 ± 4.8 ^a^	24.3 ± 4.7 ^a^	25.3 ± 4.8 ^a^
**C20:3****	53.8 ± 22.8 ^ab^	147.2 ± 32.2 ^bc^	281.7 ± 67.3 ^de^	320.7 ± 36.3 ^e^	7.7 ± 3.6 ^a^	126.1 ± 61.8 ^abc^	198.7 ± 73.7 ^cd^	115.3 ± 33.7 ^abc^	155.8 ± 30.7 ^bc^	163.7 ± 29.1 ^bc^	171.7 ± 27.4 ^bcd^	179.6 ± 25.7 ^cd^	150.9 ± 33.4 ^bc^	149.3 ± 33.2 ^bc^	147.8 ± 33.0 ^bc^	146.3 ± 32.8 ^bc^	174.6 ± 51.1 ^cd^	136.7 ± 33.7 ^bc^
**C22:5 ^E^ ****	3.5 ± 6.1^a^	109.7 ± 21.2 ^b^	nd	190.5 ± 31.1 ^c^	nd	nd	111.1 ± 18.1 ^b^	111.9 ± 18.3 ^b^	110.3 ± 21.1 ^b^	110.2 ± 20.9 ^b^	110.1 ± 20.7 ^b^	110.0 ± 20.6 ^b^	115.5 ± 21.9 ^b^	115.4 ± 21.7 ^b^	115.3 ± 21.5 ^b^	115.2 ± 21.3 ^b^	112.3 ± 19.9 ^b^	116.7 ± 20.6 ^b^
**C24:0**	60.0 ± 6.2^a^	218.3 ± 42.9^ab^	2231.6 ± 581.8	1280.4 ± 111.4 ^e^	186.0 ± 93.2 ^ab^	217.9 ± 40.0 ^ab^	329.4 ± 58.5 ^abc^	211.0 ± 48.1 ^ab^	293.3 ± 30.0 ^ab^	367.1 ± 18.2 ^bc^	441.0 ± 12.6 ^c^	514.9 ± 19.5 ^d^	231.3 ± 48.1 ^ab^	235.5 ± 51.1 ^ab^	239.6 ± 54.0 ^ab^	243.8 ± 57.0 ^ab^	276.7 ± 50.7 ^ab^	223.6 ± 47.1 ^ab^
**C22:6 ^E^ ^**	18.5 ± 17.0	47.7 ± 14.3	207.0 ± 292.7	nd	112.2 ± 112.2	22.8 ± 20.0	51.6 ± 23.3	36.6 ± 18.5	58.9 ± 24.9	69.8 ± 35.5	80.8 ± 46.0	91.8 ± 56.5	52.4 ± 12.4	55.5 ± 10.2	58.6 ± 8.8	61.7 ± 8.5	50.2 ± 15.6	43.9 ± 16.4
**Sum of EFA (g/kg)**	19.7 ± 0.7^bc^	23.3 ± 2.2^cdef^	55.3 ± 0.6 ^fgh^	36.9 ± 8.5 ^efgh^	3.0 ± 0.0 ^a^	61.0 ± 11.7 ^h^	51.1 ± 8.7 ^gh^	14.9 ± 2.6 ^b^	24.2 ± 2.4 ^cdef^	25.1 ± 2.7 ^cdefg^	25.9 ± 3.0 ^cdefg^	26.8 ± 3.4 ^defgh^	23.0 ± 2.3 ^bcde^	22.4 ± 2.3 ^bcde^	22.9 ± 1.9 ^bcde^	21.3 ± 2.2 ^bcde^	37.3 ± 5.1 ^fgh^	18.5 ± 2.4 ^bcd^
**Sum of SFA (g/kg)****	6.9 ± 0.2^abc^	9.4 ± 1.6^bcd^	30.2 ± 1.6 ^g^	21.4 ± 2.0 ^f^	1.7 ± 0.0 ^a^	15.5 ± 2.9 ^e^	15.3 ± 2.9 ^e^	6.3 ± 1.6 ^ab^	10.2 ± 1.5 ^bcde^	11.0 ± 1.5 ^bcde^	11.8 ± 1.4 ^cde^	12.6 ± 1.4 ^de^	9.4 ± 1.6 ^bcd^	9.2 ± 1.6 ^bcd^	9.0 ± 1.6 ^bcd^	8.9 ± 1.6 ^bcd^	12.4 ± 2.2 ^de^	8.0 ± 1.6 ^bcd^
**Sum of MUFA (g/kg)**	6.5 ± 0.2^bc^	6.9 ± 0.7 ^bc^	61.3 ± 6.4 ^h^	30.8 ± 3.2 ^gh^	5.2 ± 0.1 ^ab^	32.9 ± 6.2 ^gh^	24.6 ± 4.5 ^fg^	4.6 ± 0.9 ^a^	8.7 ± 0.8 ^c^	10.6 ± 0.9 ^d^	12.4 ± 1.1 ^de^	14.3 ± 1.3 ^def^	6.9 ± 0.7 ^bc^	6.8 ± 0.7 ^bc^	6.8 ± 0.7 ^bc^	6.7 ± 0.7 ^abc^	15.7 ± 2.5 ^ef^	5.8 ± 0.6 ^abc^
**Sum of PUFA (g/kg)**	19.9 ± 0.7 ^bc^	23.6 ± 2.3^cde^	46.4 ± 16.5 ^fgh^	37.5 ± 8.3 ^efgh^	3.1 ± 0.0 ^a^	61.2 ± 11.8 ^h^	51.3 ± 8.8 ^gh^	15.1 ± 2.6 ^b^	24.5 ± 2.4 ^cde^	25.4 ± 2.6 ^cdef^	26.3 ± 2.9 ^cdefg^	27.2 ± 3.3 ^defgh^	23.2 ± 2.3 ^bcde^	22.7 ± 2.3 ^bcde^	22.1 ± 2.3 ^bcde^	21.6 ± 2.3 ^bcde^	37.5 ± 5.2 ^fgh^	19.6 ± 2.3 ^bcd^

**Table A4:** Dry matter (%), protein content (%), amino acid profile (g/kg), sum of the essential amino acids (g/kg) and chitin content (%) of larvae on dry matter basis (mean ± SD), ^E^ essential amino acids for humans, * analysis performed in duplicate, nd: not determined, ** ANOVA analysis followed by Tukey post-hoc (letters in superscript), others were analyzed by Kruskal–Wallis.

	Diet 1	Diet 2	Diet 3*	Diet 4	Diet 5	Diet 6	Diet 7	Diet 8	Diet 9	Diet 10	Diet 11	Diet 12	Diet 13	Diet 14	Diet 15	Diet 16	Diet 17	Diet 18*
**Dry matter (%)**	28.3 ± 0.2	32.0 ± 0.0	nd	32.4 ± 0.1	32.6 ± 0.2	30.8 ± 0.2	23.5 ± 2.4	27.5 ± 1.0	31.6 ± 0.3	32.0 ± 0.2	32.6 ± 0.0	32.3 ± 0.2	31.2 ± 0.1	30.5 ± 0.2	31.9 ± 0.1	31.2 ± 0.3	31.2 ± 0.2	31.5 ± 0.5
**Protein content (%)**	48.3 ± 2.3 ^ef^	37.4 ± 1.2 ^a^	46.1 ± 1.9 ^def^	43.7 ± 2.3 ^cdef^	49.0 ± 1.4^f^	37.5 ± 3.0^a^	39.1 ± 0.8 ^ab^	38.8 ± 1.5 ^ab^	37.5 ± 2.7^ab^	37.5 ± 1.0^a^	43.6 ± 2.8 ^cdef^	38.4 ± 0.8 ^ab^	41.7 ± 0.4 ^bcde^	41.0 ± 0.4 ^abcd^	47.8 ± 1.9 ^def^	39.1 ± 0.9 ^ab^	39.2 ± 1.3 ^abc^	38.9 ± 1.9 ^ab^
**Arginine**	69.7 ± 4.2 ^ij^	39.6 ± 2.5 ^abc^	73.2 ± 3.0 ^j^	62.3 ± 10.5 ^fghij^	61.3 ± 8.0 ^ghij^	46.6 ± 5.3 ^abcdef^	42.7 ± 3.0 ^abcd^	44.8 ± 4.3 ^abcde^	56.1 ± 4.8 ^defghij^	46.9 ± 3.2 ^bcdefg^	52.2 ± 7.7 ^cdefhgij^	50.2 ± 3.3 ^cdefghi^	60.2 ± 2.5 ^hij^	57.1 ± 3.2 ^efghij^	53.4 ± 3.0 ^defghij^	49.7 ± 3.9 ^bcdefgh^	37.3 ± 1.4^a^	39.2 ± 3.2 ^ab^
**Alanine**	55.5 ± 6.2^i^	33.5 ± 1.8^a^	54.9 ± 8.4^hi^	37.9 ± 2.5 ^abcde^	42.5 ± 2.9 ^bcdefgh^	36.6 ± 2.0 ^ab^	39.3 ± 1.1 ^abcdefg^	38.7 ± 2.7 ^abcdef^	37.3 ± 1.2 ^abc^	42.1 ± 1.9 ^bcdefgh^	37.8 ± 1.8 ^abcd^	43.1 ± 4.2 ^cdefgh^	45.6 ± 0.6^ghi^	44.9 ± 1.5 ^fghi^	43.5 ± 1.9 ^efhgi^	42.7 ± 0.5 ^defgh^	36.7 ± 0.5 ^abc^	35.9 ± 1.8 ^ab^
**Aspartate**	45.1 ± 1.6 ^abcd^	40.9 ± 0.5 ^abc^	41.8 ± 1.2 ^abcd^	45.1 ± 5.4 ^abcd^	52.8 ± 6.3 ^cd^	39.8 ± 2.8^ab^	41.6 ± 0.6 ^abcd^	40.9 ± 1.7 ^abc^	38.9 ± 2.9^ab^	38.3 ± 0.8^a^	46.0 ± 5.7 ^abcd^	40.0 ± 1.9 ^ab^	41.8 ± 0.9 ^abcd^	41.6 ± 1.3 ^abc^	49.7 ± 1.3 ^bcd^	40.9 ± 1.2 ^abc^	51.9 ± 4.9^d^	49.4 ± 1.3 ^abcd^
**Cystine**	3.3 ± 0.5 ^fgh^	2.9 ± 0.3 ^efghj^	3.0 ± 0.4 ^fgh^	3.5 ± 0.8 ^gh^	4.2 ± 0.2 ^h^	0.4 ± 0.5 ^abcde^	0.6 ± 0.2 ^abcdefg^	0.6 ± 0.4 ^abcdef^	0.2 ± 0.0 ^ab^	0.1 ± 0.1 ^ab^	3.3 ± 0.8 ^fgh^	0.2 ± 0.2 ^abc^	1.8 ± 1.9 ^cdefgh^	0.2 ± 0.2 ^abcd^	3.4 ± 0.7 ^fgh^	0.1 ± 0.0 ^a^	2.3 ± 0.3 ^bcdefgh^	2.5 ± 0.2 ^defgh^
**Glutamate**	76.8 ± 3.1 ^e^	60.0 ± 0.8 ^abcde^	73.5 ± 3.9 ^cde^	64.4 ± 8.9 ^abcd^	82.7 ± 10.7 ^de^	58.4 ± 3.5 ^abcd^	56.1 ± 0.9^abc^	55.9 ± 1.2^abc^	55.9 ± 4.1^abc^	55.1 ± 0.5^ab^	67.7 ± 9.7 ^abcde^	57.1 ± 0.7 ^abcd^	59.5 ± 0.7 ^abcde^	59.3 ± 1.3 ^abcd^	71.6 ± 2.6 ^bcde^	58.5 ± 1.8^abcd^	54.8 ± 1.4^a^	56.6 ± 5.1^abcd^
**Glycine**	29.2 ± 1.2^f^	20.5 ± 0.9^a^	27.6 ± 0.5 ^def^	23.8 ± 1.8 ^abcd^	28.5 ± 0.1^ef^	23.2 ± 1.4 ^abcd^	22.9 ± 0.3 ^ab^	22.9 ± 1.6 ^abc^	23.4 ± 1.7 ^abcd^	25.7 ± 1.0 ^bcdef^	24.1 ± 1.2 ^abcde^	26.3 ± 2.7 ^bcdef^	25.7 ± 0.6 ^cdef^	25.3 ± 1.1 ^abcdef^	25.4 ± 0.5 ^abcdef^	24.0 ± 1.3 ^abcde^	24.1 ± 0.3 ^abcd^	23.7 ± 0.8 ^abcd^
**Histidine^E^**	19.5 ± 2.6 ^de^	14.4 ± 1.0^a^	17.6 ± 0.1 ^bcde^	16.7 ± 0.4 ^abcde^	19.7 ± 1.2^e^	14.7 ± 1.3 ^ab^	16.6 ± 0.3 ^abcde^	16.2 ± 0.6 ^abcd^	15.6 ± 1.2 ^abc^	16.7 ± 0.7 ^abcde^	17.2 ± 0.6 ^abcde^	16.6 ± 1.2 ^abcde^	17.5 ± 0.4 ^bcde^	17.9 ± 1.6 ^bcde^	18.0 ± 0.8 ^cde^	16.5 ± 0.7 ^abcd^	17.7 ± 0.1 ^cde^	17.6 ± 0.4 ^bcde^
**Hydroxylysine**	0.03 ± 0.05 ^ab^	0.6 ± 0.03 ^abcd^	0.34 ± 0.10 ^d^	0.2 ± 0.1 ^abcd^	0.3 ± 0.0 ^bcd^	6.4 ± 10.7 ^abcd^	0.5 ± 0.5 ^abcd^	0.00 ± 0.00 ^a^	0.1 ± 0.0 ^abc^	0.1 ± 0.0 ^abc^	0.1 ± 0.0 ^abcd^	0.1 ± 0.0 ^abcd^	0.1 ± 0.0 ^abcd^	0.1 ± 0.0 ^abcd^	0.1 ± 0.1 ^abcd^	0.1 ± 0.0 ^abcd^	0.1 ± 0.0 ^abcd^	0.3 ± 0.0 ^cd^
**Isoleucine^E^**	23.2 ± 0.7 ^bcde^	20.1 ± 0.5^a^	21.0 ± 1.5 ^abcd^	23.0 ± 1.7 ^abcde^	26.3 ± 2.0^e^	22.5 ± 1.3 ^abcde^	23.2 ± 1.3 ^bcde^	22.9 ± 0.5 ^abcde^	20.7 ± 1.4 ^abc^	20.6 ± 0.4 ^ab^	22.8 ± 2.0 ^abcde^	21.5 ± 1.0 ^abcd^	22.3 ± 0.5 ^abcde^	22.5 ± 0.5 ^abcde^	24.5 ± 1.1 ^cde^	21.9 ± 0.4 ^abcd^	25.2 ± 1.9 ^de^	25.0 ± 0.7 ^de^
**Leucine^E^ ****	41.0 ± 1.6^fg^	31.9 ± 0.1 ^ab^	40.1 ± 0.9 ^efg^	36.6 ± 1.7 ^bcdef^	42.5 ± 2.3^g^	32.4 ± 1.7 ^abc^	32.3 ± 0.9 ^abc^	31.1 ± 1.1^a^	35.3 ± 2.3 ^abcd^	35.4 ± 0.4 ^abcd^	36.8 ± 1.8 ^ccdef^	36.3 ± 0.3 ^bcde^	37.7 ± 0.7 ^def^	38.6 ± 1.0 ^defg^	38.1 ± 2.3 ^defg^	37.9 ± 0.6 ^defg^	37.2 ± 1.3 ^def^	37.6 ± 2.3 ^def^
**Lysine^E^**	36.9 ± 1.9^gh^	27.6 ± 0.7 ^bcdef^	35.1 ± 0.0 ^fgh^	32.1 ± 1.8 ^efg^	37.4 ± 2.1^h^	20.4 ± 17.2 ^abcde^	31.6 ± 1.5 ^cdefg^	32.2 ± 1.2 ^defg^	4.9 ± 0.4 ^abc^	4.6 ± 0.4 ^abc^	32.5 ± 1.9 ^efg^	4.6 ± 1.2 ^abc^	5.8 ± 0.4 ^abcd^	5.8 ± 1.3 ^abc^	33.2 ± 0.7 ^efg^	5.3 ± 0.6 ^abc^	1.9 ± 0.2 ^a^	2.1 ± 0.3 ^ab^
**Methionine^E^**	7.5 ± 0.5 ^f^	7.2 ± 0.5 ^ef^	6.9 ± 0.3 ^ef^	7.8 ± 2.0 ^f^	8.5 ± 2.2 ^f^	4.9 ± 1.3 ^cdef^	3.2 ± 0.6 ^bcdef^	2.9 ± 0.5 ^bcde^	0.8 ± 0.2 ^a^	1.8 ± 0.9 ^ab^	7.8 ± 2.0 ^f^	2.4 ± 0.6 ^abcd^	4.9 ± 3.2 ^cdef^	2.2 ± 0.8 ^abc^	9.0 ± 1.0 ^f^	1.6 ± 0.5 ^ab^	6.0 ± 0.8 ^def^	6.8 ± 0.1 ^ef^
**Phenylalanine^E^**	22.2 ± 1.0 ^abc^	19.7 ± 0.5 ^ab^	19.9 ± 0.1 ^abc^	20.9 ± 2.3 ^abc^	25.1 ± 2.7^bc^	17.2 ± 1.8^a^	20.8 ± 0.2 ^abc^	20.2 ± 1.1 ^abc^	21.4 ± 2.3 ^abc^	22.0 ± 0.3 ^abc^	22.9 ± 2.5 ^abc^	21.8 ± 1.9 ^abc^	22.7 ± 1.2 ^abc^	22.7 ± 1.4 ^abc^	25.3 ± 1.2 ^c^	22.6 ± 0.6 ^abc^	22.9 ± 0.8 ^bc^	23.2 ± 1.6 ^bc^
**Proline**	30.5 ± 1.6 ^cdef^	25.2 ± 1.5^a^	28.7 ± 0.7 ^abcdef^	28.8 ± 1.9 ^abcdef^	30.1 ± 1.1 ^bcdef^	25.9 ± 2.1 ^abc^	28.2 ± 0.1 ^abcde^	29.1 ± 1.6 ^abcdef^	27.5 ± 1.7 ^abcd^	28.2 ± 0.2 ^abcde^	29.5 ± 1.8 ^abcdef^	27.8 ± 0.2 ^abc^	30.9 ± 0.3 ^def^	31.1 ± 0.9 ^ef^	32.1 ± 1.7^f^	29.3 ± 0.3 ^abcdef^	28.6 ± 0.5 ^abcdef^	26.5 ± 1.2 ^ab^
**Serine**	18.8 ± 0.5 ^bcde^	15.7 ± 2.5^abcd^	18.6 ± 0.2 ^abcde^	20.2 ± 1.9 ^cde^	22.3 ± 1.3 ^de^	18.2 ± 2.0 ^abcde^	18.4 ± 1.0 ^abcde^	18.8 ± 0.7 ^abcde^	15.7 ± 1.2^ab^	15.7 ± 0.2^a^	18.6 ± 3.1 ^abcde^	16.3 ± 0.5 ^abc^	17.9 ± 0.3 ^abcde^	17.9 ± 0.3 ^abcde^	32.1. ± 1.7 ^e^	17.5 ± 0.9 ^abcd^	19.3 ± 0.7 ^bcde^	17.0 ± 1.1 ^abcd^
**Threonine^E^**	17.9 ± 1.7^ab^	17.9 ± 0.9^a^	18.6 ± 0.6^ab^	20.3 ± 1.8 ^abcd^	23.9 ± 1.9^d^	19.8 ± 1.6 ^abc^	19.9 ± 0.3 ^abc^	19.9 ± 0.9 ^abc^	19.1 ± 1.3 ^abc^	19.1 ± 0.3 ^abc^	20.2 ± 1.9 ^abcd^	19.5 ± 0.3 ^abc^	20.9 ± 0.1 ^abcd^	20.5 ± 0.6 ^abcd^	22.3 ± 0.6 ^cd^	19.9 ± 0.8 ^abc^	22.4 ± 0.9 ^bcd^	21.3 ± 0.7 ^abcd^
**Tyrosine**	29.2 ± 1.4 ^bcd^	31.6 ± 1.6 ^cde^	21.7 ± 0.0^cdef^	32.2 ± 2.9 ^bcd^	37.9 ± 3.4 ^ef^	21.2 ± 1.7^a^	29.0 ± 0.7 ^bc^	26.8 ± 0.3 ^ab^	35.2 ± 4.1 ^def^	34.7 ± 4.0 ^def^	36.2 ± 2.5 ^def^	34.1 ± 0.5 ^def^	37.8 ± 0.5 ^ef^	37.4 ± 0.3 ^ef^	40.5 ± 2.8^f^	36.6 ± 1.3 ^def^	34.1 ± 0.6 ^cdef^	35.5 ± 1.7 ^def^
**Valine^E^ ****	35.9 ± 2.3^ef^	25.5 ± 2.3^a^	33.6 ± 1.2 ^cdef^	31.2 ± 1.9 ^bcde^	37.4 ± 2.8^f^	28.1 ± 1.5 ^ab^	28.2 ± 0.4 ^ab^	27.9 ± 2.1 ^ab^	28.5 ± 1.7 ^abc^	29.8 ± 0.8 ^abcd^	30.5 ± 1.7 ^abcd^	30.4 ± 1.1 ^abcd^	32.2 ± 0.9 ^bcde^	32.7 ± 0.3 ^bcdef^	33.8 ± 1.3 ^def^	30.9 ± 0.5 ^bcde^	34.42 ± 1.9 ^def^	33.6 ± 0.4 ^cdef^
**Sum of EAA (g/kg)**	204.2 ± 10.6 ^gh^	164.4 ± 3.9 ^bcde^	193.0 ± 4.7 ^fgh^	188.6 ± 12.5 ^efgh^	220.8 ± 13.5 ^h^	159.9 ± 24.1 ^abcde^	175.9 ± 4.4 ^defg^	173.4 ± 5.3 ^cdefg^	146.4 ± 10.3 ^a^	149.9 ± 3.0 ^ab^	190.7 ± 13.6 ^fgh^	153.1 ± 4.1 ^abc^	163.9 ± 2.9 ^abcde^	163.0 ± 3.5 ^abcde^	204.0 ± 8.5 ^gh^	156.6 ± 2.0 ^abcd^	167.9 ± 7.2 ^bcdef^	167.1 ± 6.4 ^bcdef^
**Chitin content (%)**	4.2 ± 0.1 ^a^	6.2 ± 0.1 ^g^	4.3 ± 0.7 ^ab^	5.7 ± 0.2 ^cdefg^	6.0 ± 0.2 ^efg^	5.4 ± 0.6 ^abcdef^	4.7 ± 0.0 ^abc^	5.2 ± 0.4 ^abcdef^	5.6 ± 0.3 ^abcdef^	5.0 ± 0.2 ^abcd^	5.9 ± 0.1 ^defg^	5.0 ± 0.2 ^abcde^	5.3 ± 0.2 ^abcdef^	5.4 ± 0.1 ^bcde^	6.2 ± 0.3 ^fg^	5.4 ± 0.3 ^abcdef^	5.6 ± 0.4 ^bcdefg^	4.9 ± 0.2 ^abcde^

**Table A5:** Lipid content (%), fatty acid composition (mg/kg) and sum of essential fatty acids (g/kg) of the larvae on dry matter basis (mean ± SD), ^E^ssential fatty acids, * analysis performed in duplicate, SFE: saturated fatty acids, MUFA: monounsaturated fatty acid, PUFA: polyunsaturated fatty acids, nd: not detected, letters in superscript represent the Kruskal–Wallis analysis post-hoc analysis performed on the each parameters (^ indicates if no significant differences were observed).

	Diet 1	Diet 2	Diet 3	Diet 4		Diet 5		Diet 6	Diet 7	Diet 8	Diet 9	Diet 10	Diet 11	Diet 12	Diet 13	Diet 14	Diet 15 *	Diet 16	Diet 17	Diet 18	
**Lipid content (%)**	26.8 ± 0.5 ^fg^	26.5 ± 2.1 ^efg^	24.6 ± 0.7 ^cdefg^	22.3 ± 0.7 ^bc^		14.4 ± 1.3 ^a^		16.1 ± 2.6 ^ab^	28.0 ± 0.6 ^g^	26.7 ± 1.1 ^fg^	22.2 ± 1.0 ^abc^	26.4 ± 0.5 ^efg^	26.0 ± 1.4 ^defg^	25.5 ± 0.2 ^defg^	23.7 ± 0.3 ^bcde^	24.1 ± 0.5 ^cdef^	23.7 ± 1.8 ^bcdef^	23.3 ± 0.6 ^bcd^	24.6 ± 1.1 ^cdefg^	26.3 ± 2.2 ^efg^	
**C12:0**	114.8 ± 1.2 ^cd^	445.9 ± 33.1 ^h^	93.9 ± 5.7 ^ab^	76.1 ± 10.3 ^a^		205.6 ± 30.8 ^g^		nd	nd	nd	127.0 ± 5.1 ^de^	127.3 ± 9.2 ^def^	147.5 ± 10.2 ^efg^	106.6 ± 5.6 ^abc^	154.8 ± 10.1 ^fg^	152.6 ± 5.6 ^efg^	156.6 ± 14.9 ^fg^	107.1 ± 2.4 ^bc^	83.6 ± 14.7 ^ab^	92.5 ± 11.^ab^	
**C14:0**	1355.2 ± 78.9 ^de^	1007.2 ± 729.9 ^abcde^	1110.4 ± 45.1 ^abcde^	934.5 ± 89.7 ^ab^		915.5 ± 137.9 ^abc^		610.4 ± 24.9 ^a^	1115.4 ± 55.9 ^abcde^	1127.8 ± 266.9 ^abcde^	1165.6 ± 19.2 ^abcde^	1293.9 ± 109.3 ^bcde^	1282.9 ± 16.6 ^bcde^	1206.4 ± 85.4 ^abcde^	1345.3 ± 44.6 ^de^	1335.2 ± 30.1 ^cde^	1417.6 ± 170.7 ^e^	1214.3 ± 52.0 ^abcde^	1026.2 ± 191.9 ^abcd^	1064.0 ± 105.5 ^abcde^	
**C14:1**	6.7 ± 0.8 ^a^	68.5 ± 59.6 ^g^	14.8 ± 10.5 ^ab^	65.7 ± 1.8 ^bcdefg^		73.4 ± 9.4 ^cdefg^		36.5 ± 2.4 ^abcdef^	91.0 ± 10.6 ^efg^	101.2 ± 29.2 ^fg^	84.0 ± 4.1 ^efg^	94.4 ± 6.8 ^fg^	87.2 ± 25.4 ^efg^	82.0 ± 4.8 ^defg^	46.5 ± 39.4 ^abcdefg^	35.9 ± 10.8 ^abcde^	175.5 ± 152.4 ^fg^	93.5 ± 9.5 ^fg^	26.5 ± 3.3 ^abc^	26.6 ± 12.1 ^abcd^	
**C16:0**	56083.2 ± 6034.4 ^f^	44790.1 ± 4487.9 ^def^	36663.8 ± 1861.3 ^cde^	28458.6 ± 2355.3 ^abc^	25630.2 ± 4166.5 ^ab^		21091.5 ± 1320.2 ^a^		43334.0 ± 2697.3 ^def^	42112.6 ± 9732.7 ^cdef^	33507.9 ± 1529.3 ^bcd^	39150.1 ± 3136.5 ^cdef^	41568.7 ± 1154.4 ^cdef^	37105.7 ± 1887.1 ^cde^	39886.8 ± 1076.7 ^cdef^	43943.3 ± 455.4 ^ef^	38453.4 ± 957.9 ^cde^	38459.9 ± 2468.0 ^cdef^	36649.8 ± 8258.6 ^cdef^	37517.1 ± 3193.7 ^cde^
**C16:1**	1163.6 ± 33.7 ^bc^	1876.1 ± 474.2 ^c^	1408.3 ± 40.6 ^c^	1904.4 ± 404.7 ^c^		2235.1 ± 613.6 ^c^		586.0 ± 36.4 ^a^	1141.8 ± 56.1 ^bc^	836.2 ± 194.9 ^b^	2328.9 ± 1267.4 ^c^	2025.4 ± 367.3 ^c^	2486.8 ± 423.9 ^c^	1699.0 ± 157.1 ^c^	1886.5 ± 369.3 ^c^	1676.5 ± 318.1 ^c^	2362.4 ± 863.5 ^c^	1650.3 ± 182.5 ^c^	1949.9 ± 636.4 ^c^	1965.2 ± 358.5 ^c^	
**C18:0**	16853.7 ± 1982.5 ^f^	7016.3 ± 1660.7 ^cde^	14578.9 ± 517.4 ^ef^	3971.4 ± 781.8 ^a^		3739.4 ± 269.3 ^ab^		4914.6 ± 389.6 ^abc^	6619.9 ± 428.0 ^cde^	8172.6 ± 2120.7 ^cde^	5476.6 ± 260.7 ^bcd^	6184.3 ± 475.3 ^bcde^	6344.1 ± 186.3 ^cde^	5749.8 ± 172.2 ^bcd^	7016.7 ± 93.3 ^cde^	7311.3 ± 168.3 ^de^	6754.1 ± 1285.1 ^cde^	5832.8 ± 359.9 ^bcde^	5820.8 ± 1957.1 ^bcde^	5848.7 ± 616.1 ^bcde^	
**C18:1**	85203.8 ± 2053.0 ^fg^	50125.9 ± 4761.5 ^abcdef^	86063.9 ± 3265.6 ^g^	42715.1 ± 3604.0 ^abc^		38810.4 ± 5099.4 ^a^		38401.2 ± 2295.4 ^a^	63410.8 ± 3114.3 ^efg^	59229.2 ± 14337.9 ^def^	43408.3 ± 1586.2 ^abcde^	51682.2 ± 4995.4 ^bcdef^	52286.9 ± 1389.3 ^def^	53008.2 ± 4354.2 ^cdef^	46342.6 ± 1558.1 ^abcde^	50333.4 ± 1233.1 ^abcdef^	41067.7 ± 7600.5 ^abcd^	42754.4 ± 1742.1 ^ab^	48422.1 ± 10241.2 ^abcdef^	53162.5 ± 4887.4 ^def^	
**C18:2 ^E^**	55825.6 ± 1152.4 ^abcde^	56465.9 ± 5639.4 ^abcde^	66148.1 ± 1957.3 ^de^	54415.2 ± 4303.8 ^abcde^		21162.5 ± 2090.6 ^a^		43660.6 ± 2654.1^ab^	85450.6 ± 3695.0 ^f^	62016.7 ± 15350.2 ^abcde^	51519.1 ± 1069.1 ^abcd^	60888.4 ± 4676.4 ^bcde^	57479.0 ± 2283.4 ^bcde^	60989.6 ± 4520.4 ^cde^	54051.8 ± 1669.9 ^abcde^	54792.6 ± 1355.0 ^abcde^	36051.8 ± 30320.3 ^abcd^	50342.8 ± 1918.6 ^abc^	50183.6 ± 10468.0 ^abcde^	72319.7 ± 5283.8 ^e^	
**C18:3 ^E^**	2216.5 ± 32.0 ^d^	3841.4 ± 387.0 ^def^	1198.1 ± 48.5 ^b^	2672.6 ± 221.7 ^de^		1729.9 ± 177.5 ^c^		1031.3 ± 54.7 ^a^	3517.3 ± 183.5 ^def^	3904.9 ± 958.7 ^def^	3543.4 ± 101.0 ^def^	4052.8 ± 314.1 ^ef^	3546.1 ± 134.8 ^def^	3740.0 ± 274.7 ^def^	4030.8 ± 123.9 ^f^	3964.3 ± 115.9 ^ef^	2692.2 ± 2334.8 ^f^	3854.5 ± 143.5 ^ef^	3296.7 ± 699.3 ^def^	3477.8 ± 229.8 ^def^	
**C18:4 ^**	33.8 ± 23.4	nd	18.1 ± 14.2	nd		nd		nd	nd	nd	31.7 ± 0.6	30.7 ± 3.0	nd	37.9 ± 6.7	22.2 ± 19.3	22.3 ± 19.5	nd	nd	nd	nd	
**C20:0**	804.7 ± 88.2 ^ef^	398.7 ± 44.2 ^abcd^	845.3 ± 38.0 ^f^	353.8 ± 31.9 ^abc^		301.0 ± 68.0 ^a^		574.0 ± 42.1 ^bcdef^	550.8 ± 17.5 ^bcdef^	641.6 ± 201.0 ^cdef^	330.1 ± 30.8 ^ab^	433.6 ± 42.5 ^abcde^	422.4 ± 12.5 ^abcde^	443.0 ± 47.1 ^abcde^	378.6 ± 53.4 ^abcd^	422.6 ± 5.4 ^abcde^	628.9 ± 556.2 ^abcde^	315.3 ± 18.3 ^ab^	402.2 ± 111.5 ^abcde^	451.3 ± 57.2 ^abcde^	
**C20:1**	252.1 ± 3.6 ^ab^	749.0 ± 76.3 ^ij^	453.5 ± 19.7 ^cde^	870.4 ± 68.7 ^ij^		44.1 ± 38.4 ^a^		nd	506.0 ± 36.5 ^cdefg^	370.0 ± 57.9 ^bc^	524.6 ± 11.9 ^defgh^	624.1 ± 69.4 ^fghij^	931.6 ± 81.7 ^j^	649.4 ± 38.3 ^ghij^	523.0 ± 5.4 ^defgh^	544.6 ± 17.3 ^efghi^	1179.8 ± 1001.3 ^hij^	482.7 ± 22.1 ^cdef^	388.5 ± 97.7 ^bcd^	524.7 ± 97.6 ^cdefgh^	
**C20:2**	nd	287.1 ± 149.9 ^abc^	nd	478.5 ± 12.4 ^c^		144.0 ± 20.8 ^a^		313.6 ± 12.1 ^abc^	233.0 ± 83.3 ^abc^	186.3 ± 80.8 ^a^	275.0 ± 6.4 ^abc^	318.9 ± 22.5 ^abc^	459.3 ± 58.3 ^bc^	331.6 ± 27.4 ^abc^	241.1 ± 5.5 ^abc^	262.3 ± 3.6 ^abc^	279.3 ± 82.3 ^abcd^	234.5 ± 7.3 ^ab^	328.1 ± 99.4 ^abc^	375.2 ± 115.5 ^abc^	
**C20:5 ^E^**	37.5 ± 3.8 ^a^	nd	40.8 ± 1.0 ^ab^	nd		12.6 ± 21.9 ^ab^		nd	62.4 ± 108.0 ^ab^	46.3 ± 80.2 ^ab^	132.3 ± 114.6 ^ab^	244.5 ± 24.9 ^b^	nd	225.7 ± 31.5 ^ab^	129.6 ± 113.5 ^ab^	202.6 ± 15.6 ^ab^	nd	199.7 ± 16.5 ^ab^	111.6 ± 21.2 ^ab^	330.3 ± 366.2 ^ab^	
**C22:0**	144.9 ± 6.1 ^a^	622.6 ± 89.3 ^efg^	201.5 ± 7.0 ^abc^	910.9 ± 62.5 ^g^		178.2 ± 7.4 ^ab^		216.8 ± 19.7 ^abcd^	331.4 ± 8.2 ^cde^	257.8 ± 76.0 ^abcde^	nd	nd	738.1 ± 113.6 ^fg^	nd	66.4 ± 115.0 ^abcd^	nd	444.1 ± 169.2 ^def^	nd	208.1 ± 58.5 ^abcd^	307.9 ± 74.7 ^bcde^	
**C22:1**	3.5 ± 6.1 ^ab^	nd	5.7 ± 4.9 ^bc^	nd		nd		nd	nd	nd	261.9 ± 92.6 ^c^	268.1 ± 41.1 ^c^	nd	281.1 ± 9.3 ^c^	242.2 ± 21.4 ^bc^	260.3 ± 9.8 ^c^	nd	248.4 ± 6.7 ^c^	26.3 ± 7.5 ^ab^	40.0 ± 17.4 ^abc^	
**C20:4 ^**	nd	nd	nd	nd		nd		nd	nd	nd	7.4 ± 1.4	15.9 ± 7.1	nd	9.7 ± 2.7	7.8 ± 6.9	12.7 ± 1.4	nd	12.3 ± 1.2	nd	nd	
**C20:3**	nd	805.2 ± 230.1 ^cd^	nd	283.7 ± 8.2 ^ab^		240.6 ± 18.9 ^a^		213.0 ± 32.1 ^a^	432.5 ± 12.2 ^bcd^	466.0 ± 177.3 ^bcd^	456.1 ± 185.8 ^bcd^	581.5 ± 133.7 ^bcd^	548.7 ± 108.8 ^bcd^	715.9 ± 368.5 ^bcd^	559.8 ± 136.8 ^bcd^	772.9 ± 179.4 ^cd^	348.5 ± 44.7 ^bc^	760.8 ± 347.0 ^cd^	1884.9 ± 1777.3 ^d^	893.6 ± 121.0 ^d^	
**C22:5 ^E^**	nd	nd	nd	nd		17.2 ± 29.8 ^abc^		156.4 ± 137.5 ^abc^	850.5 ± 64.1 ^c^	677.0 ± 118.7 ^abc^	516.3 ± 207.9 ^abc^	675.2 ± 307.2 ^abc^	nd	800.6 ± 144.7 ^bc^	286.9 ± 313.7 ^abc^	511.2 ± 304.7 ^abc^	nd	553.6 ± 194.3 ^abc^	38.8 ± 10.2 ^a^	13.3 ± 23.0 ^ab^	
**C24:0**	15.7 ± 3.7 ^a^	164.0 ± 6.9 ^b^	88.9 ± 0.9 ^ab^	172.4 ± 27.2 ^b^		28.8 ± 25.0 ^ab^		66.5 ± 115.2 ^b^	207.5 ± 179.7 ^b^	nd	nd	nd	213.0 ± 18.2 ^b^	nd	nd	nd	311.7 ± 371.0 ^b^	nd	28.5 ± 49.4 ^ab^	35.8 ± 62.0 ^ab^	
**C22:6 ^E^ ^**	nd	9.8 ± 17.0	nd	nd		2.4 ± 4.2		nd	nd	189.7 ± 328.5	61.9 ± 13.1	129.5 ± 14.8	nd	96.4 ± 24.5	31.7 ± 54.8	83.4 ± 72.5	74.1 ± 110.5	100.1 ± 18.3	1.3 ± 2.2	nd	
**Sum of EFA (g/kg)**	58.1 ± 1.2 ^abcd^	60.3 ± 6.0 ^abcdef^	68.1 ± 2.3 ^def^	57.1 ± 4.5 ^abcd^		22.9 ± 2.3 ^a^		44.8 ± 2.7 ^ab^	89.2 ± 3.6 ^f^	66.0 ± 16.3 ^abcdef^	55.8 ± 1.3 ^abcd^	66.0 ± 5.3 ^bcdef^	61.0 ± 2.4 ^bcdef^	65.9 ± 5.0 ^cdef^	58.5 ± 1.4 ^abcde^	59.6 ± 1.2 ^abcde^	57.6 ± 1.7 ^abcd^	55.1 ± 2.1 ^abc^	53.6 ± 11.2 ^abcd^	73.0 ± 2.7 ^ef^	
**Sum of SFA (g/kg)**	75.4 ± 8.2 ^e^	54.4 ± 6.9 ^cde^	53.6 ± 2.4 ^de^	34.9 ± 2.6 ^ab^		31.0 ± 4.5 ^a^		27.5 ± 1.8 ^a^	52.2 ± 2.7 ^cde^	52.3 ± 12.4 ^bcde^	40.6 ± 1.5 ^bc^	47.2 ± 3.6 ^bcde^	50.7 ± 1.3 ^bcde^	44.6 ± 1.9 ^bcd^	48.8 ± 1.2 ^bcde^	53.2 ± 0.5 ^de^	48.2 ± 1.4 ^bcde^	45.9 ± 2.9 ^bcd^	44.2 ± 10.5 ^bcde^	45.3 ± 3.9 ^bcd^	
**Sum of MUFA (g/kg)**	86.6 ± 2.1 ^fg^	52.8 ± 5.3 ^abcdef^	87.9 ± 3.3 ^g^	45.6 ± 3.5 ^acd^		41.2 ± 5.0 ^ab^		39.0 ± 2.3 ^a^	65.1 ± 3.2 ^efg^	60.5 ± 14.6 ^bcdef^	46.6 ± 2.4 ^abcd^	54.7 ± 5.4 ^bcdef^	55.8 ± 1.5 ^def^	55.7 ± 4.5 ^def^	49.0 ± 1.3 ^abcdef^	52.9 ± 1.3 ^abcdef^	44.8 ± 7.3 ^abcd^	45.2 ± 1.9 ^abc^	50.8 ± 10.9 ^abcdef^	55.7 ± 5.3 ^cdef^	
**Sum of PUFA (g/kg)**	58.1 ± 1.2 ^abcd^	61.4 ± 6.0 ^abcdef^	67.4 ± 2.0 ^def^	57.7 ± 4.3 ^abcde^		23.3 ± 2.3 ^a^		45.4 ± 2.8 ^ab^	90.5 ± 3.7 ^f^	67.5 ± 16.7 ^abcdef^	56.5 ± 1.5 ^abcd^	66.9 ± 5.4 ^bcdef^	62.0 ± 2.5 ^bcdef^	66.9 ± 5.3 ^cdef^	59.4 ± 1.2 ^abcde^	60.6 ± 1.1 ^abcde^	39.4 ± 32.5 ^abc^	56.1 ± 2.3 ^abc^	55.8 ± 9.5 ^abcde^	77.4 ± 5.8 ^ef^	
